# Descending pathways generate perception of and neural responses to weak sensory input

**DOI:** 10.1371/journal.pbio.2005239

**Published:** 2018-06-25

**Authors:** Michael G. Metzen, Chengjie G. Huang, Maurice J. Chacron

**Affiliations:** Department of Physiology, McGill University, Montreal, Quebec, Canada; Albert Einstein College of Medicine, United States of America

## Abstract

Natural sensory stimuli frequently consist of a fast time-varying waveform whose amplitude or contrast varies more slowly. While changes in contrast carry behaviorally relevant information necessary for sensory perception, their processing by the brain remains poorly understood to this day. Here, we investigated the mechanisms that enable neural responses to and perception of low-contrast stimuli in the electrosensory system of the weakly electric fish *Apteronotus leptorhynchus*. We found that fish reliably detected such stimuli via robust behavioral responses. Recordings from peripheral electrosensory neurons revealed stimulus-induced changes in firing activity (i.e., phase locking) but not in their overall firing rate. However, central electrosensory neurons receiving input from the periphery responded robustly via both phase locking and increases in firing rate. Pharmacological inactivation of feedback input onto central electrosensory neurons eliminated increases in firing rate but did not affect phase locking for central electrosensory neurons in response to low-contrast stimuli. As feedback inactivation eliminated behavioral responses to these stimuli as well, our results show that it is changes in central electrosensory neuron firing rate that are relevant for behavior, rather than phase locking. Finally, recordings from neurons projecting directly via feedback to central electrosensory neurons revealed that they provide the necessary input to cause increases in firing rate. Our results thus provide the first experimental evidence that feedback generates both neural and behavioral responses to low-contrast stimuli that are commonly found in the natural environment.

## Author summary

Feedback input from more central to more peripheral brain areas is found ubiquitously in the central nervous system of vertebrates. In this study, we used a combination of electrophysiological, behavioral, and pharmacological approaches to reveal a novel function for feedback pathways in generating neural and behavioral responses to weak sensory input in the weakly electric fish. We first determined that weak sensory input gives rise to responses that are phase locked in both peripheral sensory neurons and in the central neurons that are their downstream targets. However, central neurons also responded to weak sensory inputs that were not relayed via a feedforward input from the periphery, because complete inactivation of the feedback pathway abolished increases in firing rate but not the phase locking in response to weak sensory input. Because such inactivation also abolished the behavioral responses, our results show that the increases in firing rate in central neurons, and not the phase locking, are decoded downstream to give rise to perception. Finally, we discovered that the neurons providing feedback input were also activated by weak sensory input, thereby offering further evidence that feedback is necessary to elicit increases in firing rate that are needed for perception.

## Introduction

Understanding how sensory information is processed by the brain in order to give rise to perception and behavior (i.e., the neural code) remains a central problem in systems neuroscience. Such understanding is complicated by the fact that natural sensory stimuli have complex spatiotemporal characteristics. Specifically, these frequently consist of a fast time-varying waveform whose amplitude (i.e., the “envelope” or contrast) varies more slowly [1–3]. Envelopes are critical for perception [4,5], yet their neural encoding continues to pose a challenge to investigators because their extraction (i.e., signal demodulation) requires a nonlinear transformation [6,7].

It is generally thought that peripheral sensory neurons implement such demodulation through phase locking, in which action potentials only occur during a restricted portion of the stimulus cycle, and that such signals are further refined downstream to give rise to perception. Indeed, in the auditory system, peripheral auditory fibers respond to amplitude-modulated sounds because of phase locking [8], with the most sensitive units displaying detection thresholds similar to those of the organism (see [3] for review). Sensitivity to amplitude modulations (AMs) increases in higher-level areas (e.g., cochlear nuclei, inferior colliculus, auditory cortex), thereby exceeding that seen at the periphery, but the underlying mechanisms remain poorly understood [3,9–12]. The common wisdom is that these are feedforward in nature and involve integration of afferent input from the sensory periphery. Here, we show that refinement of neural sensitivity to AMs that occurs in central brain areas is not due to integration of afferent input but is rather mediated by feedback pathways, thereby mediating perception and behavior.

Wave-type weakly electric fish generate a quasi-sinusoidal signal called the electric organ discharge (EOD) around their body, which allows exploration of the environment and communication. During interactions with conspecifics, each fish experiences sinusoidal AMs as well as phase modulations (PMs) of its EOD (i.e., a beat). This beat can interfere with electrolocation of other objects when the frequency is low. Specifically, such stimuli elicit a jamming avoidance response (JAR) in which both fish shift their EOD frequencies in order to increase the beat frequency to higher values that do not interfere with electrolocation. The neural circuitry giving rise to the JAR is well understood and involves feedforward integration of AM and PM information that is processed in parallel by separate neural pathways that later converge (see [13] for review), although JAR behavior can sometimes be elicited by stimuli consisting of AMs or PMs only [14]. In particular, neural sensitivities to AM and PM components increase in higher-level areas, thereby explaining the animal’s remarkable behavioral acuity [15].

Experiments focusing on the JAR have typically but not always used beats with constant depth of modulation (i.e., the envelope or contrast). More recent studies have focused on studying how time-varying contrasts, which carry information as to the distance and relative orientation between both fish [16,17], are processed by the AM neural pathway to give rise to behavioral responses that consist of the animal’s EOD frequency tracking the detailed time course of the envelope [7,18–28]. P-type peripheral electrosensory afferents (EAs) scattered over the animal’s skin surface encode EOD amplitude, but not PMs, and synapse onto pyramidal cells (PCells) within the electrosensory lateral line lobe (ELL). PCells are the sole output neurons of the ELL and project to higher brain areas that mediate behavior. Moreover, PCells receive large amounts of input from descending pathways (i.e., feedback) [29–31] that have important functional roles such as gain control [32,33], adaptive stimulus cancellation [34–41], coding of natural electro-communication signals [42], and synthesizing neural codes for moving objects [43], as well as shifting the tuning properties of PCells contingent on the stimulus’s spatial extent [44–46]. However, whether and how feedback input determines PCell responses to time-varying contrasts have not been investigated to date. Moreover, while previous studies have focused on studying neural and behavioral responses to high-contrast stimuli [7,18–28], we instead focused on low-contrast stimuli that are more commonly found in the natural environment [17].

## Results

**Fig 1:**
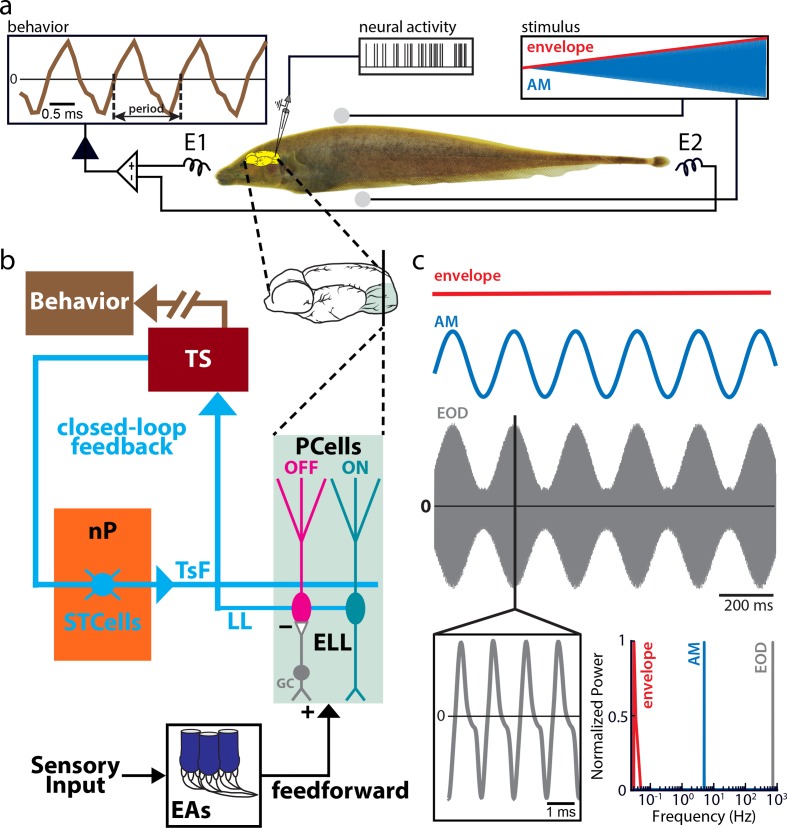
(a) Schematic of the experimental setup. The animal’s EOD (brown trace upper left) is monitored through electrodes located in front and behind the animal (E1 and E2) while the stimulus waveform (blue, upper right; the red trace shows the stimulus amplitude) is delivered using a separate set of electrodes positioned on each side of the animal (gray spheres). The stimulus was an AM of the animal’s own EOD consisting of a sinusoid with frequency 5 Hz (blue) whose amplitude (i.e., the envelope) increased linearly as a function of time (red), such that the resulting modulation depth or contrast was between 0% (no modulation) and 100% (full modulation). (b) Relevant anatomy and circuitry. EAs respond to EOD AMs and synapse onto PCells within the ELL. While ON-type PCells (turquoise) receive direct excitatory input from EAs, OFF-type PCells (magenta) receive indirect inhibitory input from EAs through local interneurons (GCs, gray). PCells are the sole output neurons of the ELL, and all project via the LL (cyan) to the midbrain TS (red). Neurons within TS project to higher brain areas that mediate the animal’s behavioral response (brown) and also feedback to STCells (cyan) within the nP (orange) that themselves project back to ELL PCells, thereby forming a closed loop. (c) Envelope (red), AM (blue), and full signal received by the animal (gray), with their respective frequency contents (bottom right). AM, amplitude modulation; EA, electroreceptor afferent; ELL, electrosensory lateral line lobe; EOD, electric organ discharge; GC, granule cell; LL, lateral lemniscus; nP, nucleus praeeminentialis; PCell, pyramidal cell; STCell, stellate cell; TS, torus semicircularis; TsF, tractus stratum fibrosum.

The goal of this study was to understand how behavioral responses to low-contrast stimuli are generated by neural circuits in the animal’s brain. To do so, we used an awake-behaving preparation in which the immobilized animal is respirated within an otherwise empty tank (Fig 1A). The animal’s behavioral response is determined from its EOD, which is being continuously recorded (Fig 1A, upper left) during stimulation (Fig 1A, upper right). The relevant neural circuitry is shown in Fig 1B. EAs make direct excitatory synaptic contact and indirect inhibitory synaptic contact with ON- and OFF-type ELL PCells, respectively. PCells project directly to torus semicircularis (TS) neurons, which in turn project to higher brain areas mediating behavioral responses. However, some TS neurons also project back to ELL via stellate cells (STCells) within the nucleus praeeminentialis (nP), thereby forming a closed feedback loop (Fig 1B, cyan). Our stimuli consisted of AMs of the animal’s own EOD that mimicked those encountered during interaction with a same-sex conspecific. Specifically, interference between the two EODs gives rise to a sinusoidal AM (Fig 1C, blue) whose depth of modulation (i.e., the envelope, red) is inversely related to the relative distance between both fish. It is important to realize that the animal’s EOD is a carrier and that the AM is the relevant stimulus here. We are considering both first- (i.e., AM) and second-order (i.e., envelope or contrast) features of the stimulus and note that these correspond to the second- and third-order features of the full signal received by the animal, respectively. Thus, the first- and second-order features correspond to the time-varying mean and variance of the stimulus, respectively. The AM, envelope, and full-signal waveforms with their respective frequency contents are shown in Fig 1C. Our stimuli consisted of a 5 Hz sinusoidal waveform (blue) whose contrast (red) increased linearly as a function of time.

### Weakly electric fish give behavioral responses to low contrasts

**Fig 2:**
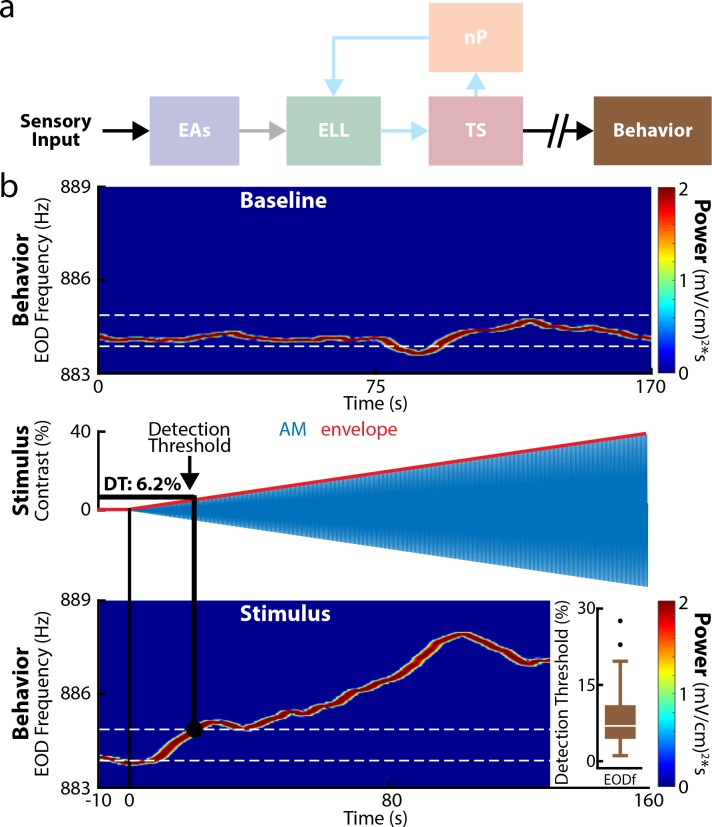
Weakly electric fish display low behavioral contrast detection thresholds. (a) Relevant anatomy diagram showing the main brain areas considered. (b) Top: EOD spectrogram (i.e., time-varying power spectrum showing frequency as a function of time) obtained under baseline conditions (i.e., in the absence of stimulation but in the presence of the animal’s unmodulated EOD). We found that the frequency at which there is maximum power (i.e., the EOD frequency) fluctuated as a function of time, which was used to compute the range of values that contains 95% of the probability distribution (white dashed lines) to determine whether behavioral responses obtained under stimulation were significantly different from those obtained in the absence of stimulation. Middle: Stimulus waveform (blue) and its envelope (red) showing contrast as a function of time. Bottom: EOD spectrogram in response to the stimulus. It is seen that the EOD frequency (“EODf”) increases after stimulus onset. The detection threshold is the contrast corresponding to the earliest time after stimulus onset for which the EOD frequency was outside the range of values determined in the absence of stimulation (black circle and white dashed line). Inset: Population-averaged detection threshold values for behavior (*n* = 35, brown). The data can be downloaded at https://figshare.com/s/93707200732db87bb80f. EA, electroreceptor afferent; ELL, electrosensory lateral line lobe; EOD, electric organ discharge; nP, nucleus praeeminentialis; TS, torus semicircularis.

We first investigated behavioral responses to increasing contrast (Fig 2A). To do so, we first quantified behavioral responses in the absence of stimulation by looking at the time-varying EOD frequency (Fig 2B, top). Plotting the EOD spectrogram (i.e., the time-varying power spectrum of the measured EOD trace) revealed that the frequency at which there was maximum power (i.e., the EOD frequency) fluctuated slightly (Fig 2B, top) around a mean value. We used these fluctuations to compute a probability distribution and to determine the interval of values that contains 95% of this distribution (Fig 2B, white dashed lines, see Materials and methods). During stimulation, we found that the animal’s EOD frequency increased more or less linearly as a function of time (Fig 2B, bottom). The detection threshold was computed as the contrast corresponding to the smallest time after stimulus onset for which the EOD frequency was outside the range of values determined in the absence of stimulation (Fig 2B, bottom, black circle and white dashed lines). We found that fish could reliably detect weak contrasts as evidenced from low detection thresholds (*n* = 35 fish, 8.8% ± 1.1%, min: 1.1%, max: 27.6%, Fig 2B, bottom, inset). The detection threshold values obtained were furthermore robust to large changes in filter settings (S1 Fig).

Our behavioral results show that electrosensory neural circuits must extract the time-varying stimulus contrast (i.e., implement signal demodulation). We thus investigated next how electrosensory neurons respond to increasing contrast.

### Peripheral EAs provide information about low contrasts through phase locking but not through firing rate

**Fig 3:**
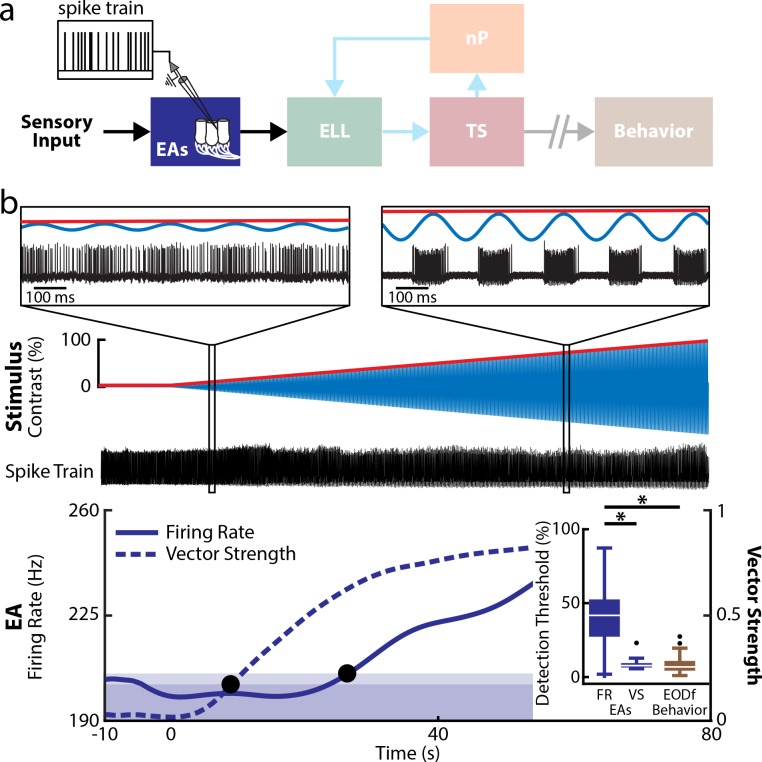
EAs reliably detect low-contrast stimuli through phase locking but not through overall changes in firing rate. (a) Relevant anatomy diagram showing the main brain areas considered. Recordings were made from individual EAs. (b) Top: Stimulus waveform (blue) and its envelope (red) showing contrast as a function of time. Middle: Spiking activity (black) from a representative example EA. The insets show magnification at two time points. In both cases, the spiking response is modulated. Bottom: Double y-axis plot showing the mean firing rate (solid blue) and VS (dashed blue) of this EA as a function of time. The bands delimit the upper range of values determined from this EA activity in the absence of stimulation for VS (dark blue) and firing rate (light blue). The detection threshold obtained from VS (leftmost black circle) was much lower than that obtained from the mean firing rate (rightmost black circle). Inset: The population-averaged detection thresholds obtained from firing rate (left) was significantly higher than those obtained from VS (middle) and behavior (right) (Kruskal-Wallis, df = 2, Firing Rate–VS: *p* = 2.6 × 10^−9^; firing rate–Behavior: *p* = 8.9 × 10^−5^). The population-averaged detection threshold obtained from VS was not significantly different than that obtained from behavior (Kruskal-Wallis, df = 2, *p* = 1). “*” indicates significance at the *p* = 0.05 level. The data can be downloaded at https://figshare.com/s/93707200732db87bb80f. EA, electrosensory afferent; ELL, electrosensory lateral line lobe; nP, nucleus praeeminentialis; TS, torus semicircularis; VS, vector strength.

We first recorded from peripheral EAs (Fig 3A). EAs are characterized by high-baseline firing rates in the absence of stimulation within the range of 200–600 spk s^−1^ [19,47]. Our dataset confirms these previous results as the baseline firing rates were all within this range (population average: 400.8 ± 18.0 spk s^−1^, *n* = 54, *N* = 5 fish). As done for behavior, we used the baseline activity of EAs to determine whether the observed neural activity was due to stimulation. We note that this is physiologically realistic as, in order to be detected, a stimulus must perturb the ongoing baseline activity of EAs. Overall, we found that EA activity was phase locked to the stimulus waveform for both low (Fig 3B, left inset) and high (Fig 3B, right inset) contrasts. Notably, for high contrasts, we observed stronger phase locking in that there was cessation of firing activity during some phases of the stimulus cycle (Fig 3B, right inset). We quantified EA responses to stimulation using standard measures of firing rate (see Materials and methods) and phase locking (i.e., the vector strength [VS], see Materials and methods). Overall, the time-varying VS quickly became significantly different from baseline (i.e., in the absence of stimulation) after stimulus onset (Fig 3B, dashed blue), leading to low phase locking detection threshold values (Fig 3B, left black circle). However, the mean firing rate (Fig 3B, solid blue) only became significantly different from baseline for larger contrasts, leading to higher firing rate detection threshold values (Fig 3B, right black circle). We note that, while there is no complete dichotomy between phase locking and firing rate, our results above do show that it is possible to increase phase locking without increasing firing rate for low contrasts.

Similar results were seen across our dataset in that EA phase locking thresholds were low and comparable to behavioral values (EA VS: 9.1% ± 1.1%, behavior: 8.8% ± 1.1%, Kruskal-Wallis, df = 2, *p* = 0.99), whereas those computed from firing rate were much higher than behavioral thresholds (38.2% ± 3.1%; Kruskal-Wallis, df = 2, p = 8.9 × 10^−5^; Fig 3B, inset). Neural detection threshold values obtained were also robust to large changes in filter settings (S1 Fig). Thus, our results show that, for low contrasts (i.e., <15%), EA firing rate modulations (i.e., phase locking) carry the information necessary to implement signal demodulation. However, such demodulation must occur downstream of EAs, as their mean firing rates were effectively unchanged relative to baseline conditions. For high contrasts (i.e., >40%), our results show that EAs implement signal demodulation, as their firing rates are then different from baseline.

### ELL PCells provide downstream brain areas information about low contrasts through both their firing rates and phase locking

**Fig 4:**
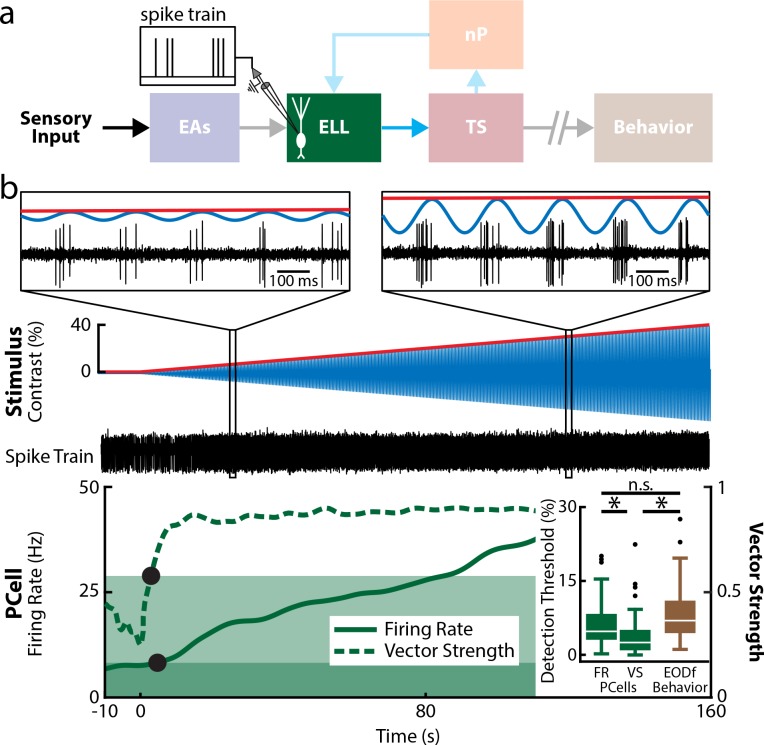
ELL PCell responses display low firing rate and phase locking detection threshold values that are comparable to behavior. (a) Relevant anatomy diagram showing the main brain areas considered. Recordings were made from individual PCells. (b) Top: Stimulus waveform (blue) and its envelope (red) showing contrast as a function of time. Middle: Spiking activity (black) from a representative example PCell. The insets show magnification at two time points. In both cases, the PCell activity strongly phase locked to the stimulus, but the average number of spikes per stimulus cycle increased with contrast. Bottom: Double y-axis plot showing the mean firing rate (solid green) and VS (dashed green) of this PCell as a function of time. The bands delimit the upper range of values determined from this PCell’s activity in the absence of stimulation. The detection threshold obtained from firing rate (rightmost black circle) was similar to that obtained from VS rate (leftmost black circle). Inset: The population-averaged detection thresholds obtained from firing rate (left) were not significantly different from behavior (Kruskal-Wallis, df = 2, *p* = 0.23). The population-averaged detection thresholds obtained from VS (middle) were significantly lower compared to those obtained from firing rate or behavior (right) (Kruskal-Wallis, df = 2, Firing Rate–VS: *p* = 0.0054; VS-behavior: *p* = 1.1 × 10^−5^). The data can be downloaded at https://figshare.com/s/93707200732db87bb80f. EA, electrosensory afferent; ELL, electrosensory lateral line lobe; EODf, electric organ discharge frequency; nP, nucleus praeeminentialis; PCell, pyramidal cell; TS, torus semicircularis; VS, vector strength.

We next recorded from the downstream targets of EAs: PCells within the ELL (Fig 4A). ELL PCells have much lower baseline firing rates than EAs, which are typically within the 5–45 Hz range [48]. Baseline firing rates of our PCell data were all within this range (population average: 13.2 ± 0.8 spk s^−1^, *n* = 59, *N* = 27 fish). We found that, like EAs, ELL PCell spiking activity was phase locked to the stimulus shortly after stimulus onset (Fig 4B, dashed green curve and Fig 4B, insets). Phase locking was seen for both low and high contrasts in that spiking only occurred during a restricted portion of the stimulus cycle (Fig 4B, compare left and right panels). However, PCells responded in a qualitatively different fashion than EAs in that their firing rates also became significantly different from baseline shortly after onset (Fig 4B, solid green curve). Thus, firing rate detection threshold values for PCells were comparable to those found for behavior (PCells firing rate: 7.0% ± 0.9%; Kruskal-Wallis, df = 2, *p* = 0.23; Fig 4B, bottom inset), whereas phase locking detection thresholds for PCells were significantly lower than firing rate and behavioral detection thresholds (VS: 3.9% ± 0.6%; Kruskal-Wallis, df = 2, Firing Rate–VS: *p* = 0.0054; VS-Behavior: *p* = 1.1 × 10^−5^; Fig 4B, bottom inset). We further tested the relationship between neural and behavioral detection thresholds by plotting values obtained from neurons in different individual fish. Overall, there was a strong correlation between neural and behavioral detection threshold values (S2A Fig, *n* = 10, *N* = 10 fish, 3 repetitions each; r = 0.93; *p* = 4.6 × 10^−7^), indicating that neurons with low detection thresholds were primarily found in fish with low behavioral detection thresholds. There was, however, no correlation between the trial-to-trial variabilities of neural and behavioral detection thresholds to repeated stimulus presentations (S2B Fig, *n* = 10, *N* = 10 fish, 3 repetitions each; r = −0.14; *p* = 0.48), indicating that fluctuations in the activity of a single ELL PCell do not significantly influence behavior. Overall, our results show that, for low contrasts (i.e., <15%), PCell phase locking and firing rate both carry information about contrast. As such, either phase locking or firing rate could be decoded by downstream brain areas in order to give rise to behavior.

### Feedback input to PCells causes increases in firing rate, while feedforward input causes increased phase locking for low contrasts

**Fig 5:**
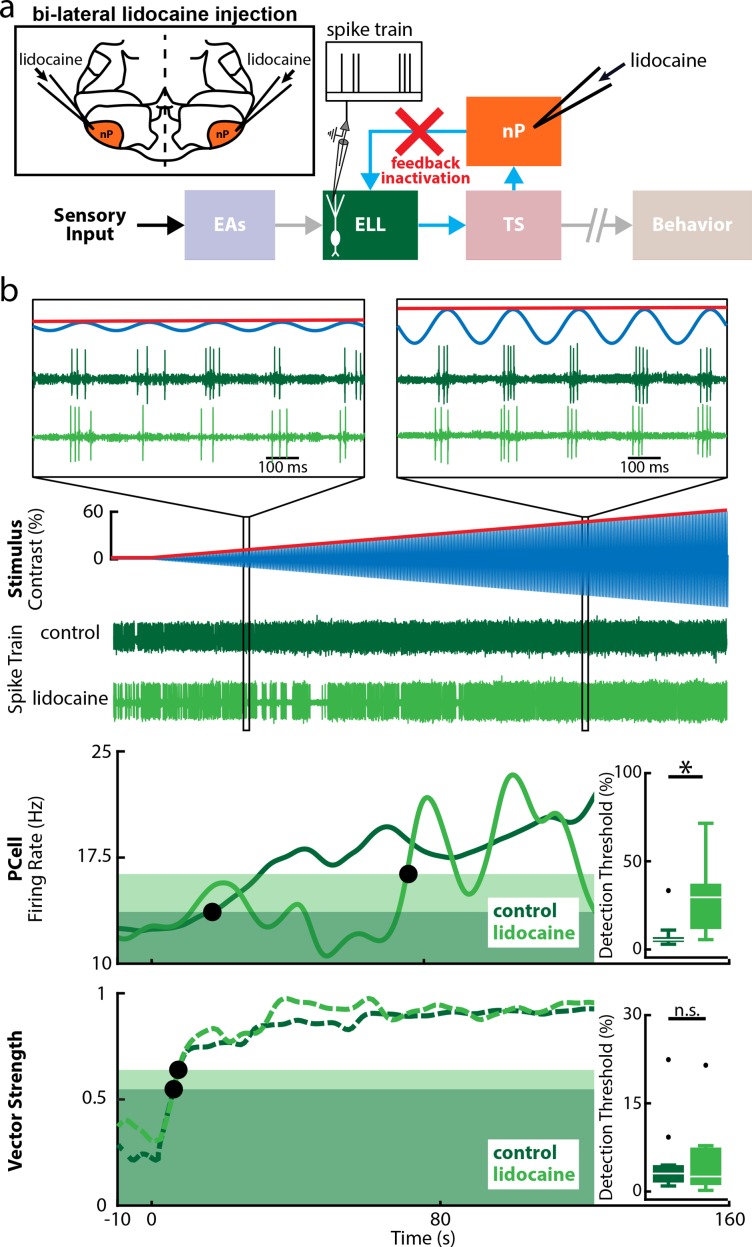
Feedback inactivation strongly increases PCell detection thresholds computed from firing rate but does not affect those computed from phase locking. (a) Relevant anatomy diagram showing the main brain areas considered. Recordings were made from individual PCells while lidocaine, a sodium channel antagonist, was injected bilaterally into nP (top left inset), which will inactivate feedback input (red cross). (b) Top: Spiking activity of a representative PCell before (dark green) and after (light green) feedback inactivation, in response to the stimulus (blue) and its time-varying envelope (red) as a function of time. The insets show magnification at two time points. PCell activity was strongly phase locked to the stimulus before and after feedback inactivation, indicating a strong response to feedforward input from EAs even for low-stimulus contrasts. Middle: Mean firing rates before (solid green) and after (light green) feedback inactivation. The detection threshold of this cell strongly increased after feedback inactivation (compare the position of the leftmost and rightmost black circles). Inset: The population-averaged firing rate detection thresholds were significantly increased after feedback inactivation (Wilcoxon sign rank test, *p* = 0.0039). Bottom: Vector strength curves as a function of time before (solid green) and after (light green) feedback inactivation for this same cell. The phase locking detection thresholds (black circles) before and after feedback inactivation were similar to one another. Inset: The population-averaged phase locking detection threshold was not significantly altered by feedback inactivation (Wilcoxon sign rank test, *p* = 0.92). The data can be downloaded at https://figshare.com/s/93707200732db87bb80f. EA, electrosensory afferent; ELL, electrosensory lateral line lobe; nP, nucleus praeeminentialis; n.s., not significant; PCell, pyramidal cell; TS, torus semicircularis.

Perhaps the simplest explanation for why PCells phase lock to stimuli with low contrasts is that they simply linearly integrate feedforward input from EAs, which are already phase locked to these. What then causes PCells to increase their firing rates in response to stimuli with low contrasts? Unlike the explanation above for increased phase locking, this cannot be due to linear integration of feedforward input from EAs. This is because our results show that, for low contrasts, EA firing rates are not significantly different from baseline values. One possibility is that increases in PCell firing rate result from nonlinear integration (e.g., half-wave rectification) of feedforward input from EAs. Another possibility is that increases in firing rate are due to feedback input. To determine the relative roles of feedforward and feedback inputs, we pharmacologically inactivated all feedback input onto ELL PCells by injecting lidocaine, a sodium channel antagonist, bilaterally into nP (*n* = 10 cells, *N* = 4 fish, see Materials and methods, Fig 5A). Importantly, this manipulation does not alter feedforward input onto ELL PCells, since EAs do not receive feedback input. Thus, if increases in firing rate are due to feedforward input, then we would expect that PCell responses will be relatively unaffected and that the firing rate detection threshold will remain the same as under control conditions. If, on the other hand, increases in firing rate are due to feedback input, then we would expect that, after complete feedback inactivation, PCells will no longer respond to low-contrast stimuli through increases in firing rate, thereby significantly increasing the firing rate detection threshold.

We found that complete feedback inactivation strongly altered ELL PCell responses to stimuli with increasing contrast (Fig 5B). Indeed, PCell firing rate only became significantly different from baseline for much higher contrasts (Fig 5B, middle, compare dark and light solid green curves). Consequently, PCell firing rate detection threshold values were much higher after feedback inactivation (control: 8.4% ± 2.9%; lidocaine: 29.7% ± 6.3%, Fig 5B, middle inset). We note that this was not due to changes in the integration of feedforward input, as phase locking was unaffected (Fig 5B, bottom, compare dark and light dashed green curves). Indeed, phase locking threshold values were similar before and after complete feedback inactivation (control: 5.2% ± 2.1%; lidocaine: 5.1% ± 2.0%, Fig 5B, bottom inset). We note that vehicle injection (i.e., saline) did not affect ELL PCell firing rate (*n* = 7, *N* = 3 fish, control: 9.4% ± 1.0%; saline: 9.3% ± 0.7%, S3 Fig) or phase locking (control: 5.4% ± 1.7%; saline: 4.8% ± 1.5%, S3 Fig) detection thresholds. Thus, while increases in PCell firing rate were no longer observed for low (<15%) contrasts after complete feedback inactivation, such inactivation did not affect phase locking. These results show that it is possible to alter firing rate without altering phase locking. We conclude that, during low-contrast stimulation, increased PCell firing rate is due to feedback input, while increased phase locking is instead due to feedforward input from EAs.

### Changes in PCell firing rate, but not phase locking, determine behavioral responses

**Fig 6:**
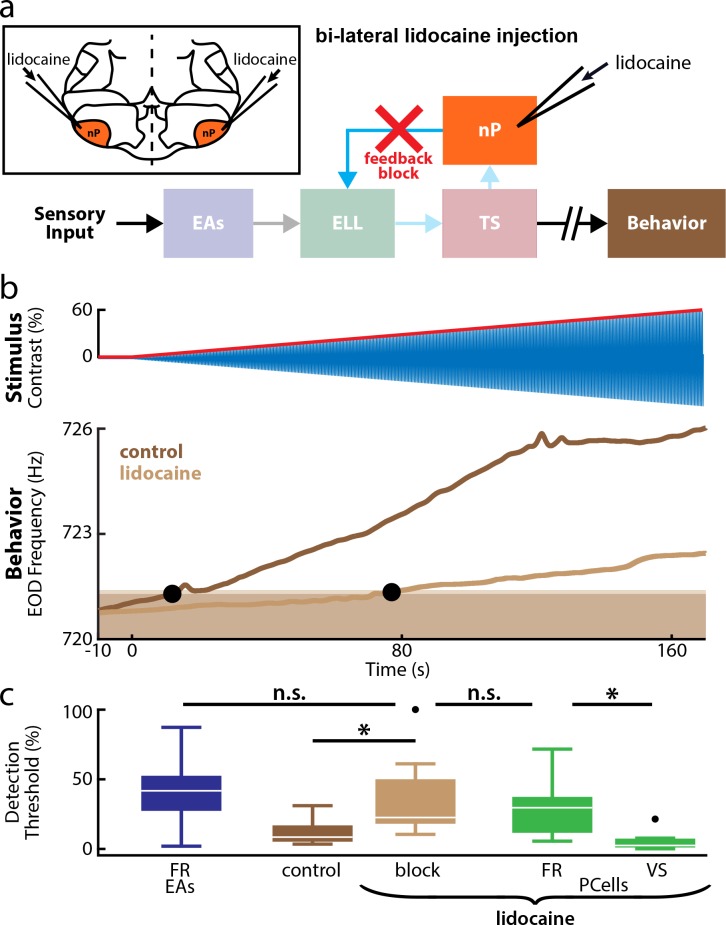
Feedback inactivation strongly increases behavioral detection thresholds. (a) Relevant anatomy diagram showing the main brain areas considered. Behavioral responses were recorded before and after lidocaine, a sodium channel antagonist, was injected bilaterally into the nPs (top left inset), which will inactivate feedback input onto ELL PCells (red cross). (b) Top: Stimulus waveform (blue) and its envelope (red) showing contrast as a function of time. Bottom: EOD frequency from a representative example individual fish before (dark brown) and after (light brown) feedback inactivation. The behavioral detection threshold strongly increased after feedback inactivation (compare the position of the leftmost and rightmost black circles). (c) Whisker-box plots comparing population-averaged detection thresholds computed from behavior before (dark brown) and after (light brown) feedback inactivation to those obtained from PCells after feedback inactivation computed using firing rate (green, left) and phase locking (green, right) and to those computed from firing rate in EAs (blue). Overall, PCell firing rate was a much better predictor of behavior than phase locking. The similarity of detection thresholds obtained from EA and PCell firing rate to that of behavior after feedback inactivation strongly suggests that, for low contrasts (<15%), phase locking in EAs is detected by PCells but is not decoded by downstream brain areas to give rise to behavior. “*” indicates significance at the *p* = 0.05 level using a Wilcoxon sign rank or Kruskal-Wallis test (behavior). The data can be downloaded at https://figshare.com/s/93707200732db87bb80f. EA, electrosensory afferent; ELL, electrosensory lateral line lobe; EOD, electric organ discharge; nP, nucleus praeeminentialis; n.s., not significant; PCell, pyramidal cell; Ts, torus semicircularis; VS, vector strength.

Our results so far show that the increase in PCell firing rate shortly after stimulus onset (i.e., to low contrasts) is due to feedback, while increased phase locking is instead due to feedforward input from EAs. In theory, either PCell firing rate or phase locking could be used to determine behavioral responses. If the former, then increases in EOD frequency shortly after stimulus onset are due to increases in PCell firing rate. If the latter, then nonlinear integration of PCell input by downstream neurons would give the information necessary to drive behavior. To test which of PCell firing rate or phase locking is relevant for determining behavior, we investigated how complete feedback inactivation affected behavioral responses, as this manipulation does not affect ascending pathways from TS to higher brain areas mediating behavior (Fig 6A). On the one hand, if phase locking is necessary to elicit behavior, then we would expect that feedback inactivation will not affect behavioral responses to low contrasts and thus that behavioral detection threshold values will be largely unaffected. On the other hand, if changes in PCell firing rate are necessary to elicit behavioral responses, then we would expect that feedback inactivation will cause cessation of behavioral responses to low contrasts, thereby increasing the behavioral detection threshold.

We found that complete feedback inactivation gave rise to significant changes in behavioral responses (*N* = 15 fish). Indeed, behavioral responses to low contrasts (<15%) were no longer present, as the EOD frequency remained below the response level (Fig 6B, compare light and dark brown curves). EOD frequency only became significantly different from baseline for much larger contrasts than under control conditions, leading to significantly larger behavioral detection threshold values (control: 12.2% ± 2.3%; lidocaine: 34.2% ± 6.3%, Wilcoxon sign rank test, p = 6.1 × 10^−5^, Fig 6C, brown boxes). We note that vehicle injection (i.e., saline) did not significantly affect behavioral detection thresholds (*N* = 10 fish, control: 13.9% ± 1.5%; saline: 14.1% ± 1.9%, S3 Fig). Thus, our results show that, for low contrasts, the information carried by PCell phase locking is not decoded by downstream areas to determine behavior. Rather, it is the increase in PCell firing rate that is necessary to elicit behavioral responses. Interestingly, complete feedback inactivation increased PCell firing rate and behavioral detection thresholds to values that were similar to those obtained for the firing rate of single EAs (Fig 6C). Thus, not only do our results show that feedforward input from EAs is sufficient to elicit changes in PCell firing rate for high (>40%) contrasts, but they also suggest that it is the changes in EA firing rate that are then necessary to elicit behavioral responses to high contrasts, rather than phase locking.

### Closed-loop direct feedback input mediates changes in ELL PCell firing rate

ELL PCells receive two sources of feedback input. One source originates directly from nP and forms a closed loop with ELL PCells, while the other instead originates indirectly from nP and goes through the eminentia granularis posterior (EGP) (S4 Fig). To test which pathway mediates changes in PCell firing rate responses, we performed two additional manipulations. The first was selectively blocking the indirect pathway by injection of 6-cyano-7-nitroquinoxaline-2,3-dione (CNQX) within the ELL, which did not significantly alter PCell firing rate or phase locking, as well as behavioral responses (S4 Fig). The second was to selectively inactivate direct feedback by injecting lidocaine unilaterally within the TS, which gave rise to similar changes in PCell activity as those observed with complete feedback inactivation (compare S5 Fig to Fig 5). Thus, these results show that it is closed-loop feedback that causes increases in PCell firing rate in response to low-contrast stimuli. We will return to this point in the Discussion section.

### nP STCells providing direct feedback input to ELL PCells increase their firing rates with increasing contrast

**Fig 7:**
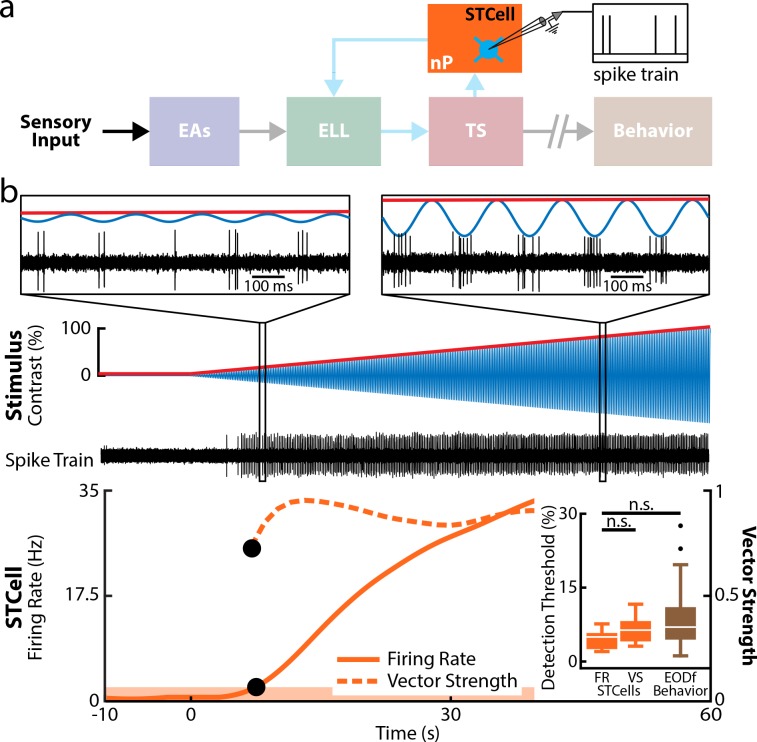
STCells within nP display low detection thresholds that are comparable to behavior. (a) Relevant anatomy diagram showing the main brain areas considered. Recordings were made from individual STCells. (b) Top: Stimulus waveform (blue) and its envelope (red) showing contrast as a function of time. Middle: Spiking activity from a representative STCell. The insets show magnification at two time points. In both cases, the STCell activity strongly phase locked to the stimulus, but the average number of spikes per stimulus cycle increased with contrast, similar to that observed for PCells under control conditions. Bottom: Double y-axis plot showing the mean firing rate (solid orange) and VS (dashed orange) of this STCell as a function of time. The band delimits the upper range of values determined from this STCell’s firing rate in the absence of stimulation. Because STCells tended to not fire action potentials in the absence of stimulation, it was not possible to compute a threshold level for the VS. The VS detection threshold (upper black circle) was thus set to the lowest contrast for which the STCell reliably fired action potentials for at least 5 consecutive stimulus cycles (see Materials and methods). Inset: The population-averaged detection thresholds obtained from firing rate (left) and VS (middle) were not significantly different from one another or from behavior (Kruskal-Wallis, df = 2; Firing Rate–VS: *p* = 0.51; Firing Rate–Behavior: *p* = 0.06; VS-Behavior: *p* = 0.99). The data can be downloaded at https://figshare.com/s/93707200732db87bb80f. EA, electrosensory afferent; ELL, electrosensory lateral line lobe; EODf, electric organ discharge frequency; nP, nucleus praeeminentialis; PCell, pyramidal cell; STCell, stellate cell; VS, vector strength.

How does closed-loop feedback input enable increases in PCell firing rate for low-stimulus contrasts? To answer this question, we recorded from nP STCells (*n* = 10, *N* = 3 fish) that provide direct feedback input to ELL PCells in response to increasing contrast (Fig 7A). We used previously established criteria [30] to identify STCells (S6 Fig). Overall, STCells were mostly silent in the absence of stimulation and started firing shortly after stimulus onset (Fig 7B, bottom, solid orange line). Overall, their firing rate detection thresholds were comparable to those of PCells under control conditions as well as behavior (STCells: 4.6% ± 0.7%, min: 2.0%, max: 7.6%; Fig 7B, inset). We also found that STCells phase locked to the stimulus at the onset of firing (Fig 7B, bottom, dashed orange line). Consequently, their phase locking detection thresholds were also low (6.5% ± 0.9%; Fig 7B, inset). Thus, our results show that STCells, by increasing their firing activity in response to low-contrast stimuli, provide the necessary input to drive increases in ELL PCell firing rates.

### Summary

**Fig 8:**
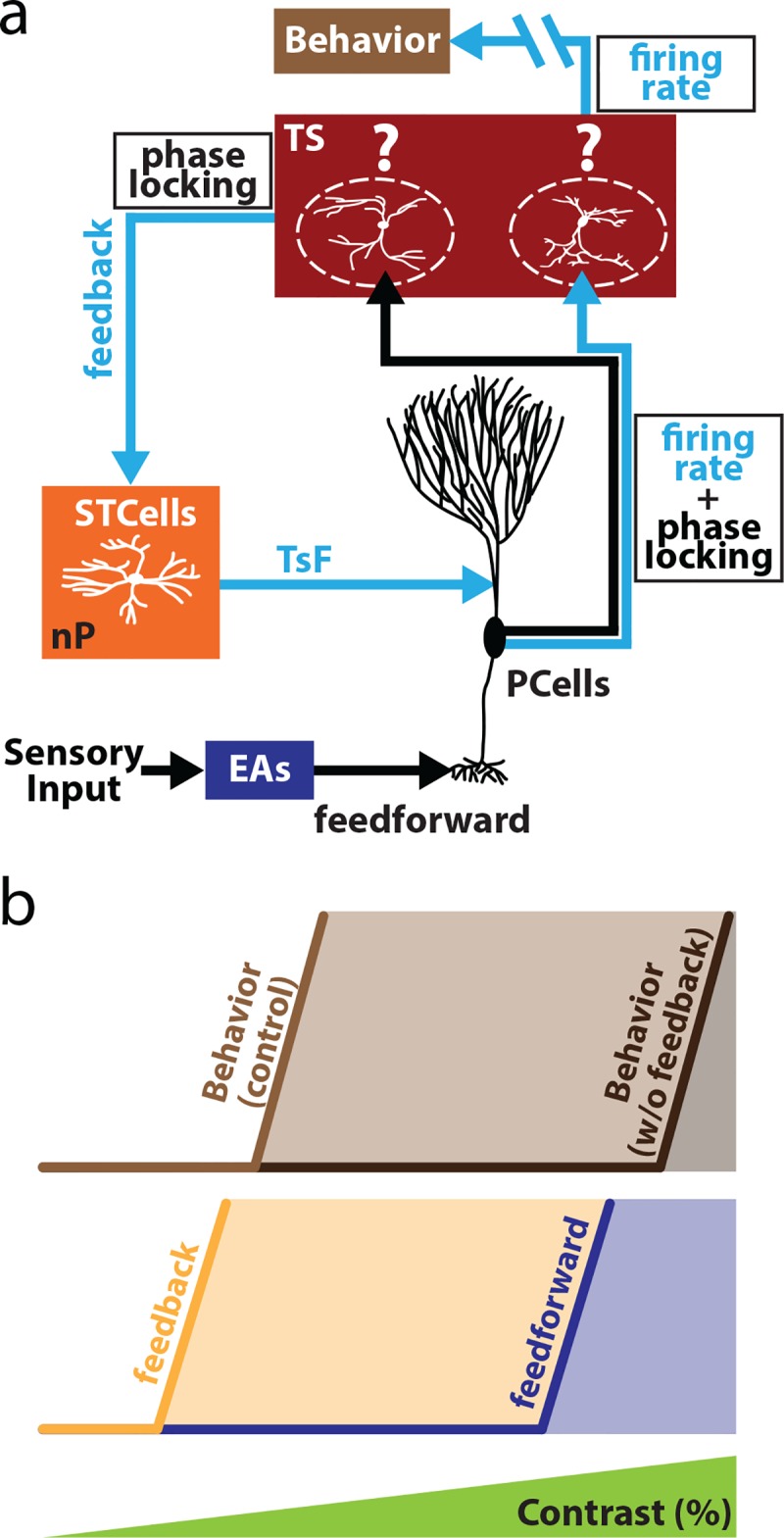
Summary of results. (a) Relevant circuitry showing ELL PCells receiving feedforward input from EAs and projecting to TS. It is assumed that some neurons within TS decode information carried by PCell firing rate and in turn project to higher brain areas to give rise to behavior. It is further assumed that a separate group of neurons within TS project back to ELL via nP STCells and receive phase locking information from PCells, thereby forming the closed feedback loop that is necessary to elicit increases in firing rate and behavioral responses to low contrasts. (b) Summary of the relative contributions of feedback and feedforward inputs toward determining behavioral responses as a function of contrast. For low contrasts (<15%) and under control conditions, feedback is necessary to elicit changes in PCell firing rate and behavior. However, for higher (>40%) contrasts, feedforward input is sufficient to elicit increases in PCell firing rate and behavior. This is because our results show that, after complete feedback inactivation, both PCell and behavior tended to be only elicited for contrasts for which EAs increased their mean firing rates. EA, electrosensory afferent; ELL, electrosensory lateral line lobe; nP, nucleus praeeminentialis; PCell, pyramidal cell; STCell, stellate cell; TS, torus semicircularis.

Fig 8 shows the proposed contributions of feedforward and feedback inputs toward determining behavioral responses to increasing stimulus contrast. Overall, EAs phase lock to low contrasts, which causes ELL PCells to in turn phase lock to these. While the information carried by PCell phase locking is necessary to extract the contrast (i.e., implement signal demodulation), our results show that this information is not directly decoded by downstream brain areas to give rise to behavior (Fig 8A). Rather, PCell phase locking is integrated via a closed feedback loop that is necessary to elicit increases in PCell firing rate for low contrasts, which in turn elicit behavioral responses. For high contrasts, and in the absence of feedback, our results suggest that it is changes in EA firing rate that are carried over to ELL PCells, which in turn elicit behavioral responses (Fig 8B).

## Discussion

### Summary of results

Here, we investigated how weakly electric fish process and perceive stimuli with different contrasts. Contrary to previous studies, we focused specifically on behavioral responses and the underlying neural mechanisms to low (<15%) contrasts. We found that behavioral detection thresholds were low on average (9%). Overall, peripheral EAs responded through phase locking and thus transmitted the necessary information to extract contrast to downstream areas. However, changes in EA firing rate were only elicited for much higher (approximately 40%) contrasts. ELL PCells receiving input from EAs responded to low contrasts through both increased phase locking as well as firing rate: the detection threshold values computed from either were lower than those for EAs (7% and 4%, respectively) and matched behavior (9%). Pharmacological inactivation of feedback input revealed that, while such input was necessary to elicit increases in firing rate for low contrasts, increases in phase locking were caused by feedforward input from EAs. Analysis of behavioral responses after feedback inactivation revealed that it was changes in PCell firing rate and not phase locking that determined behavior. Finally, we recorded from nP STCells that provide direct feedback input to PCells. STCells increase their firing activity shortly after stimulus onset and thus displayed low detection thresholds (5%) that matched those of PCells and behavior under control conditions. Our results thus provide the first experimental evidence showing that feedback is necessary to give rise to neural and behavioral responses to weak sensory input that would not be detected otherwise.

### Direct feedback generates neural responses to low contrasts

Our results show that behavioral and ELL PCell firing rate responses to low contrasts are generated because of closed-loop feedback. These results have strong implications for the electrosensory system as well as other systems, as described below. We note that information about low-stimulus contrast is carried by PCell phase locking and is due to feedforward input from EAs and does not require feedback. Theoretical studies have shown that it is possible to directly extract this information (e.g., by performing a nonlinear operation such as half-wave rectification followed by low-pass filtering [6]). However, our results show that downstream brain areas that mediate behavior do not decode information carried by PCell phase locking. This is because we showed that feedback inactivation strongly increased behavioral thresholds but did not alter PCell phase locking. We also note that some EAs displayed firing rate detection thresholds (approximately 2%) that are lower than those obtained at the organismal level (approximately 9%). The input from these EAs could theoretically be used to elicit behavioral responses to low contrasts. Moreover, as EAs display negligible correlations between their baseline firing rate variabilities [49–51], it is then theoretically possible to improve the signal-to-noise ratio (SNR) by linearly integrating their activities [52]. Anatomical studies have shown that the PCells considered here receive input from many (600–1,400) EAs [53], which should give rise to substantial improvement in SNR, according to theory. However, it is unlikely that the lower firing rate thresholds of PCells are due to either selectively responding to input from the most sensitive EAs or to improving the SNR. This is because our results show that, under complete feedback inactivation, PCell firing rate threshold values were similar to those of single EAs (40%). We hypothesize that this is because heterogeneities within the EA population counteract the potential beneficial effects of summing afferent activities. Indeed, previous studies have shown that EAs display large heterogeneities, particularly in terms of their baseline firing rates [47], which can strongly influence how they respond to envelopes [19].

It is well known that ELL PCells receive both direct and indirect sources of feedback [29]. However, the functional role of the direct pathway has remained largely unknown until recently [43]. Indeed, previous studies have hypothesized that this pathway could act as a sensory searchlight that enhances salient features of sensory input, as originally hypothesized by Crick [54]. Our results provide the first experimental evidence that such feedback serves to generate sensory neural responses and perception of behaviorally relevant features of sensory input that would otherwise not be processed in the brain, which is in line with this hypothesis. In particular, nP STCells providing direct feedback input to ELL PCells have firing properties that are ideally suited for detecting low contrasts. Indeed, these cells display little to no spiking activity in the absence of stimulation [30], which is unlike ELL PCells [55,56] or multipolar cells that instead give rise to indirect feedback input onto ELL PCells [31]. Our results suggest that it is the increase in firing rate of STCells that is likely needed to increase PCell firing rate to low contrasts, thereby eliciting behavioral responses. We note that our results show that feedback plays an active role in generating increases in PCell firing rate, rather than changing how they integrate feedforward input. This is because PCell phase locking was unaffected by feedback inactivation, strongly suggesting that the response to feedforward input is similar under both conditions. This novel function for feedback is thus quite different than previously uncovered functions for feedback input such as gain control [32]. We further note that our stimuli consisting of a sinusoidal waveform whose amplitude increases with time will roughly mimic the spatially diffuse AM stimulation caused by a looming conspecific [16]. The resulting stimulation is quite different than that caused by a looming object (e.g., a prey), which instead gives rise to spatially localized stimulation consisting of changes in EOD amplitude with no envelope. However, we note that a spatially localized envelope would be also generated if an oscillating motion would be superimposed on top of the looming motion. A previous study has shown that the direct feedback pathway played an important role in determining neural responses to receding objects [43], strongly suggesting that responses to looming objects are primarily determined by feedforward input. Here, we have instead shown that the direct feedback pathway generates neural and behavioral responses to stimuli mimicking a looming conspecific. While previous studies have shown that lateral motion can give rise to changes in EOD frequency [14], how looming objects affect EOD frequency should be the focus of future studies.

An important question pertains to how feedback generates increased PCell firing rate responses to low contrasts. Such studies will require recording from the TS neurons that receive input from ELL PCells and project back to nP STCells. Previous studies have shown that there are about 50 cell types within the TS [57,58] that display highly heterogeneous responses to electrosensory stimulation [59–65]. In particular, some cell types in TS (i.e., so-called ON-OFF neurons) respond selectively to stimulus contrast because of balanced input from ON- and OFF-type ELL PCells [24]. Specifically, these neurons respond to both increases and decreases in the stimulus and are thus ideal to generate behavioral responses. This is because they will simply increase their firing rates with increasing contrast (see [7] for review). Other cell types within TS respond to contrast in a manner similar to that of ELL PCells (i.e., through changes in phase locking and firing rate) [24]. We hypothesize that it is these latter neurons that project back to nP and provide input to STCells. It is, however, important to note that all previous studies of TS neural responses used high contrasts. As such, future studies that are beyond the scope of this paper are needed to understand how different cell types within TS respond to the low contrasts considered here and mediate both ELL PCell and behavioral responses to these. Such studies should focus on brain areas downstream of TS, where it is expected that variability in the responses of single neurons would correlate with behavior, as observed in other sensory modalities [66].

What is the relationship between our observed behavioral responses to stimuli with time-varying contrasts and those previously observed using stimuli with constant contrasts? Previous studies have focused on studying the JAR behavior in response to stimuli with constant contrast. In particular, the JAR and the underlying neural circuitry have been extensively studied in the weakly electric fish species *Eigenmannia virescens* [13]. This species shows exquisite sensitivity to AM stimuli, as these generate behavioral responses with contrasts as low as 0.1% [14]. The JAR behavior in *Apteronotus* is less sensitive than for *Eigenmannia* [67], which is most likely due to the fact that the former species is less gregarious than the latter [68–70]. Further, there are important differences between the JAR behavior as well as the underlying neural circuitry in *Apteronotus* and *Eigenmannia* that have been reviewed extensively [71–73]. Most notably, *Apteronotus* tend to always increase their EOD frequency in response to low-frequency jamming stimuli with constant amplitude, which does not require the presence of PMs [74,75]. Specifically, the EOD frequency will rise and saturate to a higher value. What is the role of feedback input onto ELL PCells in determining the JAR behavior? Previous studies have shown that lesioning both indirect and direct feedback onto ELL PCells increases their phase locking responses to low-frequency sinusoidal stimuli for high but not for low contrasts [32]. Further studies have shown that this effect was mediated primarily, if not exclusively, by the indirect feedback pathway [34,45]. Our results showing that selectively blocking the direct pathway does not alter phase locking in ELL PCells are consistent with these. Although the effects of complete feedback inactivation on the JAR have, to our knowledge, not been tested in *Apteronotus*, manipulations that enhanced phase locking by ELL PCells to low-frequency stimuli also led to an enhanced JAR (i.e., a greater increase in EOD frequency) [76]. We thus predict that complete feedback inactivation would enhance the JAR and that this would be primarily, if not exclusively, due to the indirect pathway. If true, then this would imply that the role of feedback in determining JAR behavior in response to stimuli with constant contrast and our observed behavioral responses to stimuli with time-varying contrasts are qualitatively different. While the indirect feedback pathway is involved in determining the JAR magnitude via gain control, we have instead shown here that the direct feedback pathway is necessary in order to elicit increases in ELL PCell firing rate that in turn elicit increases in EOD frequency. It is nevertheless possible that the direct pathway could play a role in generating the initial increase in EOD frequency during the JAR, or in setting the latency. Further studies are needed to test these predictions.

Finally, our results show that feedback is only necessary to generate neural and behavioral responses to low contrasts. Indeed, our results show that EAs will change their firing rates for high (>40%) contrasts, which are then sufficient to elicit changes in PCell firing rate and, in turn, behavioral responses. An important question is thus: why generate responses to low contrasts through feedback when such responses could, in theory, be generated by feedforward pathways? To answer this question, one must first consider that the sinusoidal stimuli with different contrasts considered here, while behaviorally relevant, are by no means the only behaviorally relevant stimuli that must be encoded by the electrosensory system. For example, prey stimuli [77] as well as intraspecific communication stimuli [78] must also be encoded. Secondly, one must consider the actual mechanism by which EAs can encode contrast, which involves static nonlinearities (e.g., rectification and/or saturation) during which the firing activity is constant and thus cannot encode sensory input. Thus, responses to envelopes in EAs comes at a cost. This is because these neurons then cannot respond as well to other sensory input, as the firing rate is constant (either at zero or at its maximum value) for some portion of the stimulus. We thus hypothesize that generating responses to low contrast at the level of feedback pathways does not compromise ELL PCell responses to other behaviorally relevant sensory input (e.g., caused by prey). While further studies are needed to test this hypothesis, we note that ELL PCells display large heterogeneities, with some PCells receiving much less feedback than others [34,55]. It is also conceivable that these latter PCells, which also project to higher brain areas, help mediate perception of other behaviorally relevant stimuli.

### Implications for other sensory systems

Processing of AMs is behaviorally relevant in multiple sensory modalities (auditory: [3]; visual: [79]; vestibular: [50,80]; somatosensory: [81,82]). As mentioned above, AMs found in natural auditory stimuli (e.g., speech) are particularly necessary for perception [4,5]. There exist important parallels between processing of amplitude-modulated stimuli in both the auditory and electrosensory systems. Our results show that behavioral detection thresholds in weakly electric fish (approximately 9%) are similar to those found in the auditory system (approximately 4%) [83–85]. The processing of amplitude-modulated sounds by the auditory system has been extensively studied. In particular, single peripheral auditory fibers will respond to AMs because of phase locking [8] with the most sensitive neurons displaying detection thresholds that are similar to perceptual values [83] (see [3] for review). Sensitivity to AMs also increases in higher-level areas (e.g., cochlear nuclei, inferior colliculus, auditory cortex) [3,9–12]. Thus, it has been commonly assumed in the auditory system that the lower detection thresholds seen centrally are the result of integration of afferent input from the periphery, as predicted from mathematical models [86,87]. We hypothesize that the lower detection thresholds seen in more central areas are instead due to feedback. Further studies investigating the effects of feedback onto central auditory neurons are needed to validate this hypothesis.

Finally, we note that it is frequently assumed that behavioral responses are determined by feedforward integration of afferent input [66,88,89]. However, anatomical studies in several systems have shown that feedback projections from higher centers often vastly outnumber feedforward projections from the periphery [90–93], and a recent review has highlighted the need for further studies focusing on the role of feedback projections in determining how sensory information gives rise to behavioral responses [94]. Previous studies have demonstrated that feedback is involved in predictive coding [95–97] (see [98] for review) or combined with feedforward input in order to amplify neuronal responses [99]. Instead, we provide here the first experimental evidence that closed-loop feedback actually generates responses to and perception of weak or low-intensity sensory input. Our results are thus timely in that they show for the first time how feedback pathways mediate sensory neural responses to and perception of behaviorally relevant stimulus features. Important commonalities between the electrosensory system and the visual, auditory, and vestibular systems of mammals (see [100,101] for review) suggest that similar mechanisms will be found in these systems as well.

## Materials and methods

### Ethics statement

All animal procedures were approved by McGill University’s animal care committee and were performed in accordance with the guidelines of the Canadian Council on Animal Care under protocol 5285.

### Animals

The wave-type weakly electric fish *A*. *leptorhynchus* was used exclusively in this study. Animals of either sex were purchased from tropical fish suppliers and were housed in groups (2–10) at controlled water temperatures (26–29°C) and conductivities (300–800 μS cm^−1^) according to published guidelines [102].

### Surgery

Surgical procedures have been described in detail previously [44,50,103]. Briefly, 0.1–0.5 mg of tubocurarine (Sigma) was injected intramuscularly to immobilize the fish for electrophysiology and behavioral experiments. The fish was then transferred to an experimental tank (30 cm × 30 cm × 10 cm) containing water from the animal’s home tank and respired by a constant flow of oxygenated water through their mouth at a flow rate of 10 mL min^–1^. Subsequently, the animal’s head was locally anesthetized with lidocaine ointment (5%; AstraZeneca, Mississauga, ON, Canada), the skull was partly exposed, and a small window was opened over the recording region (hindbrain for ELL or midbrain for nP).

### Stimulation

The EOD of *A*. *leptorhynchus* is neurogenic and therefore is not affected by injection of curare. All stimuli consisting of AMs of the animal’s own EOD were produced by triggering a function generator to emit 1 cycle of a sine wave for each zero crossing of the EOD, as done previously [104]. The frequency of the emitted sine wave was set slightly higher (30 Hz) than that of the EOD, which allowed the output of the function generator to be synchronized to the animal’s discharge. The emitted sine wave was subsequently multiplied with the desired AM waveform (MT3 multiplier; Tucker Davis Technologies), and the resulting signal was isolated from the ground (A395 linear stimulus isolator; World Precision Instruments). The isolated signal was then delivered through a pair of chloridized silver wire electrodes placed 15 cm away from the animal on either side of the recording tank perpendicular to the fish’s rostro-caudal axis. Depending on polarity, the isolated signal either added or subtracted from the animal’s own discharge. It is important to realize that these stimuli mimic the EOD AMs but not the PMs generated during encounters with conspecifics. This is not an issue here, as these FMs do not elicit responses from the neurons considered here. Further, previous studies have shown that the behavioral responses considered here (see below) do not require PMs [28].

In order to obtain behavioral and neural (periphery: EAs; hindbrain: PCells; midbrain: STCells) detection thresholds, we used a stimulus consisting of either a 5 Hz sinusoidal or a 5–15 Hz noise (fourth-order Butterworth) carrier waveform whose depth of modulation computed with respect to the animal’s unperturbed EOD amplitude increased from 0% to 100%. We found that EA detection thresholds were similar for both sinusoidal (*n* = 15) and noisy (*n* = 39) stimulus waveforms (Kruskal-Wallis, df = 2, *p* = 0.11). Thus, detection threshold values for EAs were pooled. We only used the 5 Hz sinusoidal waveform for determining detection thresholds for ELL PCells, nP STCells, and behavior. We characterized each ELL PCell as either “ON” or “OFF” type using a noisy AM stimulus with frequency content of 0–120 Hz, as done previously [25,105]. In this case, the standard deviation of the AM was adjusted as in previous studies [51,76,106,107], as measured using a small dipole placed close to the animal’s skin in the middle of the animal’s rostro-caudal and dorsoventral axes (typically 0.2 mV cm^−1^). We note that it is likely that some of the variations in threshold values obtained for EAs are due to the location of the pore on the animal’s skin relative to the stimulus.

### Pharmacology

The composition of the vehicle/control saline was as follows (all chemicals were obtained from Sigma): 111 mM NaCl, 2 mM KCl, 2 mM CaCl_2_, 1 mM MgSO_4_, 1 mM NaHCO_3_, and 0.5 mM NaH_2_PO_4_. The pH of the saline solution was 6.8. Glutamate (Sigma), lidocaine (Astra Pharmaceuticals), and CNQX (Sigma) was dissolved in saline before application, as done previously [23]. Drug application electrodes were made using two-barrel KG-33 glass micropipettes (OD 1.5 mm, ID 0.86 mm, A-M Systems) and pulled by a vertical micropipette puller (Stoelting) to a fine tip and subsequently broken to attain a tip diameter of approximately 5 μm for each barrel. The two barrels were used for separate application of either lidocaine (1 mM) or CNQX (1 mM), as well as glutamate (1 mM) or saline. During ELL recordings for which the EGP indirect feedback was blocked with CNQX, we first used excitatory responses to glutamate application to confirm that we were within proximity of the pyramidal neuron we were recording from, as done previously [76]. CNQX was then applied to the neuron to ensure a local effect. Complete feedback inactivation was achieved by inserting 2 pipettes containing lidocaine bilaterally into nP. In order to block the direct feedback from the midbrain area TS, we performed unilateral injections of lidocaine on the contralateral TS while recording from PCells within the ipsilateral ELL. Injection locations were guided by the *Apteronotus* brain atlas [108] and determined based on somatotopic mappings. We inserted a glass pipette (20–30 μm tip) and pressure injected lidocaine at a few depths between 1,000–1,500 μm with 4–5 puffs each at a pressure of 15–20 psi and 130 ms of injection time, as done previously [43]. We note that this manipulation also blocks ascending input to higher-order brain areas mediating behavior. As such, we did not investigate the effects of injecting lidocaine within TS on behavioral responses. For behavioral recordings, injections of lidocaine, saline, and CNQX were performed bilaterally in nP and ELL, respectively, as done previously [23,76]. All pharmacological injections were performed using a duration of 130 ms at 15–20 psi using a Picospritzer (General Valve). Indirect feedback inactivation was assessed by comparing the baseline firing rates of PCells before and after drug application, as shown in a previous study [55].

### Recording

Sharp glass micropipette electrodes (20–40 MΩ) backfilled with 3 M KCl were used to record in vivo from EAs within the deep fiber layer of ELL, as described in previous studies [20,49,109]. EAs can be easily identified based on their high baseline (i.e., in the absence of stimulation) firing rates as well as from the fact that their probability of firing increases with increasing EOD amplitude [47,110]. The recording electrode was advanced into the ELL with a motorized microdrive (IW-711; Kopf). We used well-established techniques to perform extracellular recordings with Woods metal electrodes from PCells [111] located within the lateral segment of the ELL based on recording depth and mediolateral placement of the electrode on the brain surface, as done previously [21,48,105].

Similarly, we performed extracellular recordings with Woods metal electrodes from STCells in nP. STCells were confirmed based on the recording depth as well as their low spontaneous firing rate and response-tuning curves to sinusoidal AMs based on previous characterization (see S5 Fig) [30]. All recordings were digitized at a 10 kHz sampling rate using CED 1401 plus hardware and Spike2 software (Cambridge Electronic Design) and stored on a computer hard disk for offline analysis.

### Behavior

Animals were immobilized by an intramuscular injection of 0.1–0.5 mg tubocurarine and set up in the recording tank, similarly to the method described in the Recording section. Depending on which feedback pathway was pharmacologically inactivated, different surgeries were performed. Briefly, to inactivate the nP direct feedback pathway, both sides of the midbrain were exposed rostrally to T0 [108], and double-barrel pipettes containing saline and lidocaine were inserted into the nP (1,000–1,250 μm). To inactivate the EGP indirect feedback pathway, both sides of the hindbrain ELL were exposed to the caudal-lateral edge, where pipettes containing CNQX were inserted superficially (100–400 μm). Multiple injections (typically 3–5) were performed to ensure that both hemispheres of nP and ELL were sufficiently affected by the pharmacological agents. Stimuli were then presented as in the Recording section in order to elicit behavioral responses. The animal’s behavior was recorded through a pair of electrodes located at the rostrum and tail of the animal. The fish’s time-varying EOD frequency was extracted either by computing a spectrogram of the recorded signal or from the zero-crossings of the recorded EOD signal. For the former, the EOD frequency was then determined as the frequency with the highest power near the fourth harmonic of the fish’s baseline EOD frequency, and the extracted frequency was then divided by 4 in order to get the true EOD frequency of the fish. For the latter, the zero-crossings were used to generate a binary sequence (as described in the Recording section) that was low-pass filtered (second-order Butterworth filter with 0.05 Hz cutoff frequency) to obtain the time-varying EOD frequency. Quantitatively similar results were obtained using either methodology.

### Data analysis

All data analysis was performed offline using custom-written codes in MATLAB software (MathWorks). The recorded membrane potentials were first high-pass filtered (100 Hz; eighth-order Butterworth). Spike times were defined as the times at which the signal crossed a given threshold value from below. A binary sequence *R(t)* was then constructed from the spike times of each P-unit in the following manner: time was first discretized into bins of width *dt* = 0.1 ms. The value of bin *i* was set to 1 if there was a spike at time *t*_*j*_ such that *i* × *dt* < *t*_*j*_ < (*i* + 1) × *dt* and to 0 otherwise. Note that since the bin width *dt* is smaller than the absolute refractory period of the neuron, there can be at most 1 spike time that can occur within any given bin. The firing rates were obtained by filtering the binary sequence using a second-order Butterworth filter with 0.05 Hz cutoff frequency. Both neural and behavioral response detection threshold values to the stimulus were characterized by the intensity at which the firing rate or EOD frequency first became significantly different at the *p* = 0.05 level from those observed in the absence of stimulation. Specifically, the threshold was determined as the intensity for which either the neural or behavioral response was first outside the range of values that contains 95% of the probability distribution in the absence of stimulation. We note that changing the percentage value did not alter the qualitative nature of our results (S7 Fig) (Kruskal-Wallis, df = 2, Behavior: *p* = 0.99; EAs: *p* = 0.99; PCs: *p* > 0.66).

#### Time-varying VS

To determine the degree of a neuron’s phase locking to the AM stimulus, we computed a time-varying VS until the end of stimulation was reached. Therefore, spike trains were accumulated as cycle histograms, and the response was quantified using the VS *(r)*, which measures the degree of phase locking and ranges between 0 (random spiking) and 1 (perfect phase locking) [112]. VS is defined as

$$r=\frac{1}{N}\sqrt{{\left(\sum_{i}\cos\;\theta_{i}\right)}^{2}+{\left(\sum_{i}\sin\;\theta_{i}\right)}^{2}},$$ (1)

in which *N* is the number of spikes during 1 cycle of stimulation. The time-varying VS was computed by averaging the VS *r* over a time window *T* of 1 s:

$$VS=\frac{\sum_{i}r_{i}}{T},$$ (2)

in which *r*_*i*_ is the VS obtained during 1 cycle of stimulation, and *T* is a time window of 1 s (i.e., 5 cycle periods). The Rayleigh statistics (*r*^*2*^*N ≥ 3*.*5*) were used to determine significance. Varying the time window length between 0.6 and 2 s did not alter our results significantly.

#### Correlation between neural and behavioral detection thresholds

The correlation between detection threshold values of PCells and behavior obtained simultaneously during repetitive stimulation (*n* = 10, *N* = 10 fish, 3 repetitions each) was assessed using the Pearson’s correlation coefficient. The correlation between the residuals were computed by first subtracting the mean detection threshold value obtained for PCells and behavior over the 3 repetitions.

### Statistics

Statistical significance was assessed through a nonparametric Kruskal-Wallis test with Bonferroni correction or Wilcoxon sign rank test for paired measures at the *p* = 0.05 level. Values are reported as box plots unless otherwise stated. Error bars indicate ± SEM. On each box, the central mark indicates the median, and the bottom and top edges of the box indicate the 25th and 75th percentiles, respectively. The whiskers extend to the most extreme data points not considered outliers, and the outliers are plotted individually using the “•” symbol.

## Supporting information

S1 Fig Filter settings do not affect detection threshold values obtained for EAs and behavior. Detection thresholds as a function of filter cutoff frequency for EAs (blue) and behavior (brown). Light blue and light brown data points indicate the values obtained for a cutoff frequency of approximately 0.05 Hz, as used in this study. Detection thresholds did not differ significantly for different filter cutoff frequencies (EAs: Kruskal-Wallis, df = 12, *p* = 0.999 with Bonferroni correction; behavior: Kruskal-Wallis, df = 12, *p* = 0.6162 with Bonferroni correction). The data can be downloaded at https://figshare.com/s/93707200732db87bb80f. EA, electrosensory afferent.
(TIF)

S2 Fig Neuronal and behavioral detection threshold values are strongly correlated but not their residuals. (a) The detection threshold values of PCells and behavior are strongly positively correlated, as indicated by a high r-value (Pearson’s correlation coefficient: r = 0.93; *p* = 4.6 × 10^−7^) The inset shows a whisker box of the correlation coefficient obtained for each pair. (b) The residuals of neuronal and behavioral detection threshold values obtained for repetitive stimulation (3 repetitions) were not significantly correlated (Pearson’s correlation coefficient: r = −0.14, *p* = 0.48). The data can be downloaded at https://figshare.com/s/93707200732db87bb80f. PCell, pyramidal cell.
(TIF)

S3 Fig Saline injection into nP does not affect neuronal and behavioral detection thresholds. (a) Relevant anatomy diagram showing the main brain areas considered. Recordings were made from individual PCells. (b) Left: PCell firing rate detection threshold values did not change after saline injection (control: 9.4% ± 1.0%, saline: 9.3% ± 0.7%; Wilcoxon sign rank test, *n* = 7; *p* = 0.94). Middle: PCell VS detection threshold values did not change after saline injection (control: 5.4% ± 1.7%, saline: 4.8% ± 1.5%; Wilcoxon sign rank test, *n* = 7; *p* = 0.59). Right: Behavioral detection threshold values did not change after saline injection (control: 13.9% ± 1.5%, saline: 14.1% ± 1.9%; Wilcoxon sign rank test, *n* = 10; *p* = 0.99). “ns” indicates no significant difference. The data can be downloaded at https://figshare.com/s/93707200732db87bb80f. nP, nucleus praeeminentialis; PCell, pyramidal cell; VS, vector strength.
(TIF)

S4 Fig Inactivating the indirect feedback pathway does not alter detection thresholds observed for either central electrosensory neurons or from the organism. (a) Relevant anatomy diagram showing the main brain areas considered. Recordings were made from individual PCells. Right inset: bilateral injection of the non-NMDA glutamate receptor antagonist CNQX near the apical dendrites of ELL PCells in the molecular layer near the cell being recorded from, as well as to test the effects on behavior. (b) Top: Example behavioral responses to increasing contrast (top) before (dark brown) and after (light brown) bilateral CNQX injection. Middle: Example firing rate responses to increasing contrast from an example ELL pyramidal neuron (dark green) and after (light green) bilateral CNQX injection. Bottom: Example time-varying VS responses to increasing contrast from the same ELL pyramidal neuron (dark green) and after (light green) bilateral CNQX injection. We found that both behavioral (top, inset, control: 2.6% ± 0.4%; CNQX: 2.5% ± 0.4%, Wilcoxon sign rank test, *n* = 7, *p* = 0.81) and neural (firing rate: middle inset, control: 8.5% ± 3.2%; CNQX: 8.1% ± 3.0%, Wilcoxon sign rank test, *n* = 8, *p* = 0.31; VS: bottom inset, control: 3.8% ± 1.5%; CNQX: 3.2% ± 1.7%, Wilcoxon sign rank test, *n* = 8, *p* = 0.55) detection thresholds were not affected by CNQX injections. Note that previous studies have shown that saline injection within the molecular layer does not affect behavioral responses [23,76,113]. As a positive control, we note that injection of CNQX significantly decreased the baseline (i.e., in the absence of stimulation) firing rates of ELL PCells (control: 12.75 ± 1.98 spk s^−1^; CNQX: 6.79 ± 1.18 spk s^−1^, Wilcoxon sign rank test, *n* = 8, *N* = 3 fish, *p* = 0.0078), which is consistent with previous results[55,114]. “ns” indicates no significant difference. The data can be downloaded at https://figshare.com/s/93707200732db87bb80f. CNQX, 6-cyano-7-nitroquinoxaline-2,3-dione; ELL, electrosensory lateral line lobe; NMDA, N-methyl-D-aspartate; PCell, pyramidal cell; VS, vector strength.
(TIF)

S5 Fig Inactivating the direct feedback pathway by injecting lidocaine into TS significantly increases firing rate detection thresholds but does not affect phase locking detection thresholds. (a) Relevant anatomy diagram showing the main brain areas considered. Lidocaine was injected in TS while recordings were made from individual PCells within the contralateral ELL. (b) Top: Example firing rate responses to increasing contrast (top) from an example ELL pyramidal neuron (dark green) and after (light green) lidocaine injection. Bottom: Example time-varying VS responses to increasing contrast (top) from the same ELL pyramidal neuron (dark green) and after (light green) unilateral lidocaine injection into the contralateral TS. We found that firing rate detection thresholds significantly increased after lidocaine application (middle inset, control: 3.6% ± 0.5%; lidocaine: 13.6% ± 4.9%, Wilcoxon sign rank test, *n* = 12, *N* = 5 fish, *p* = 4.88 × 10^−4^). In contrast, VS detection thresholds were not significantly altered by lidocaine injections into the contralateral TS (bottom inset: control: 2.4% ± 0.7%; Lidocaine: 2.1% ± 0.7%, Wilcoxon sign rank test, *n* = 12, *p* = 0.42). We note that these results are qualitatively similar to those obtained by injecting lidocaine into nP and thereby blocking STCells (compare with Fig 5). “ns” indicates no significant difference. The data can be downloaded at https://figshare.com/s/93707200732db87bb80f. ELL, electrosensory lateral line lobe; nP, nuclus praeeminentialis; PCell, pyramidal cell; STCell, stellate cell; TS, torus semicircularis; VS, vector strength.
(TIF)

S6 Fig Identifying nP STCells based on previous characterization. Response profile of our nP STCell population (*n* = 10) to different sinusoidal AM frequencies. The firing rate modulation peaks around 4–8 Hz and is negligible for AM frequencies >32 Hz. This is similar to that reported previously for STCells [30] and strongly differs from properties of other neuron types within nP [31]. The data can be downloaded at https://figshare.com/s/93707200732db87bb80f. AM, amplitude modulation; nP, nucleus praeeminentialis; STCell, stellate cell
(TIF)

S7 Fig Detection threshold values are similar for different significance criterion. No significant differences were seen for behavior (brown, left), EAs (blue, middle), and PCells (green, right) when altering the significance level (Kruskal-Wallis, df = 2; Behavior: *p* = 0.99; EAs: p = 0.99; PCells: *p* > 0.66 with Bonferroni correction). The data can be downloaded at https://figshare.com/s/93707200732db87bb80f. EA, electrosensory afferent; PCell, pyramidal cell.
(TIF)

## Abbreviations

AM: amplitude modulation
CNQX: 6-cyano-7-nitroquinoxaline-2,3-dione
EA: electrosensory afferent
EGP: eminentia granularis posterior
ELL: electrosensory lateral line lobe
EOD: electric organ discharge
GC: granule cell
JAR: jamming avoidance response
LL: lateral lemniscus
nP: nucleus praeeminentialis
PCell: pyramidal cell
PM: phase modulation
SNR: signal-to-noise ratio
STCell: stellate cell
TS: torus semicircularis
TsF: tractus stratum fibrosum
VS: vector strength
