# Supplementary figures and images for: Descending pathways generate perception of and neural responses to weak sensory input
Source: PLoS Biol. 2018 Jun 25;16(6):e2005239. doi: 10.1371/journal.pbio.2005239 (PMC6040869; doi:10.1371/journal.pbio.2005239)

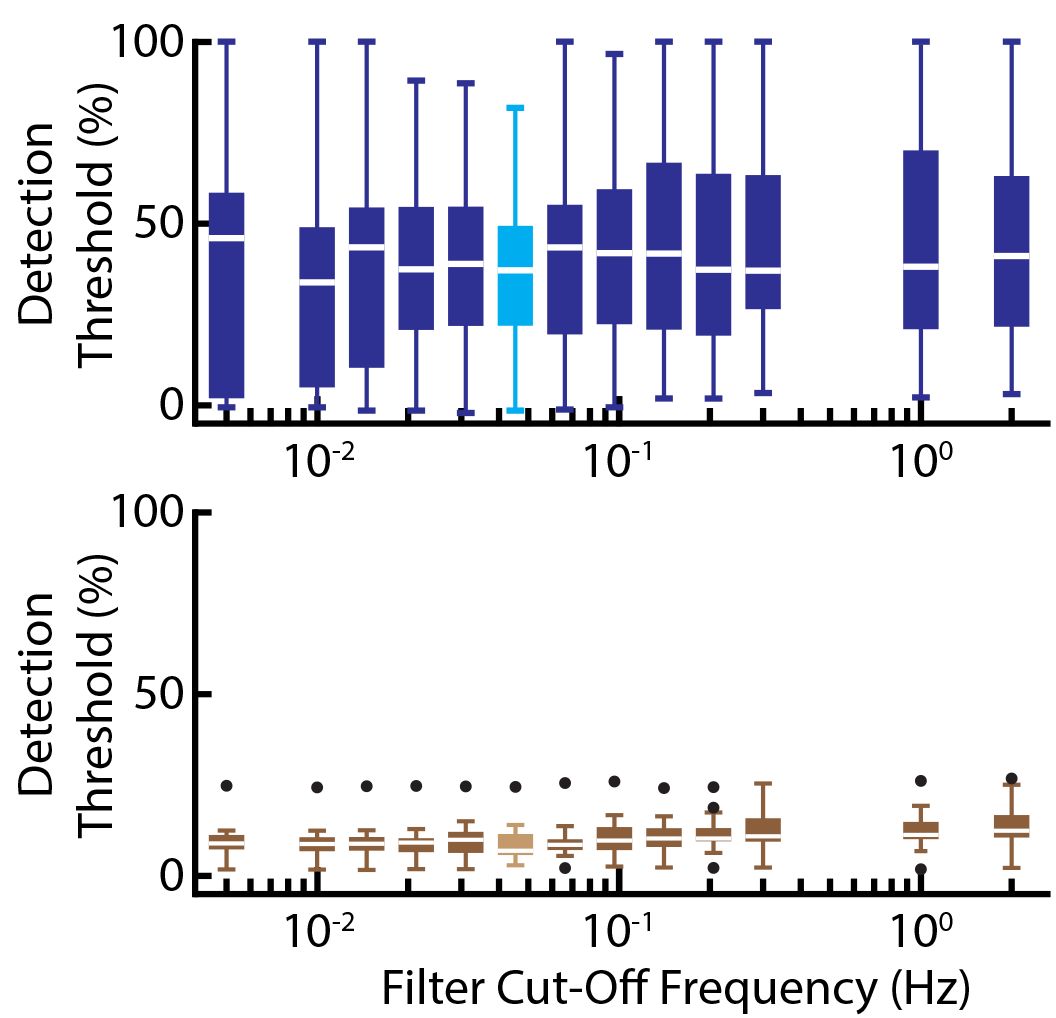

Supplement: S1 Fig — Detection thresholds as a function of filter cutoff frequency for EAs (blue) and behavior (brown). Light blue and light brown data points indicate the values obtained for a cutoff frequency of approximately 0.05 Hz, as used in this study. Detection thresholds did not differ significantly for different filter cutoff frequencies (EAs: Kruskal-Wallis, df = 12, p = 0.999 with Bonferroni correction; behavior: Kruskal-Wallis, df = 12, p = 0.6162 with Bonferroni correction). The data can be downloaded at https://figshare.com/s/93707200732db87bb80f. EA, electrosensory afferent. (TIF) [file pbio.2005239.s001.tif]

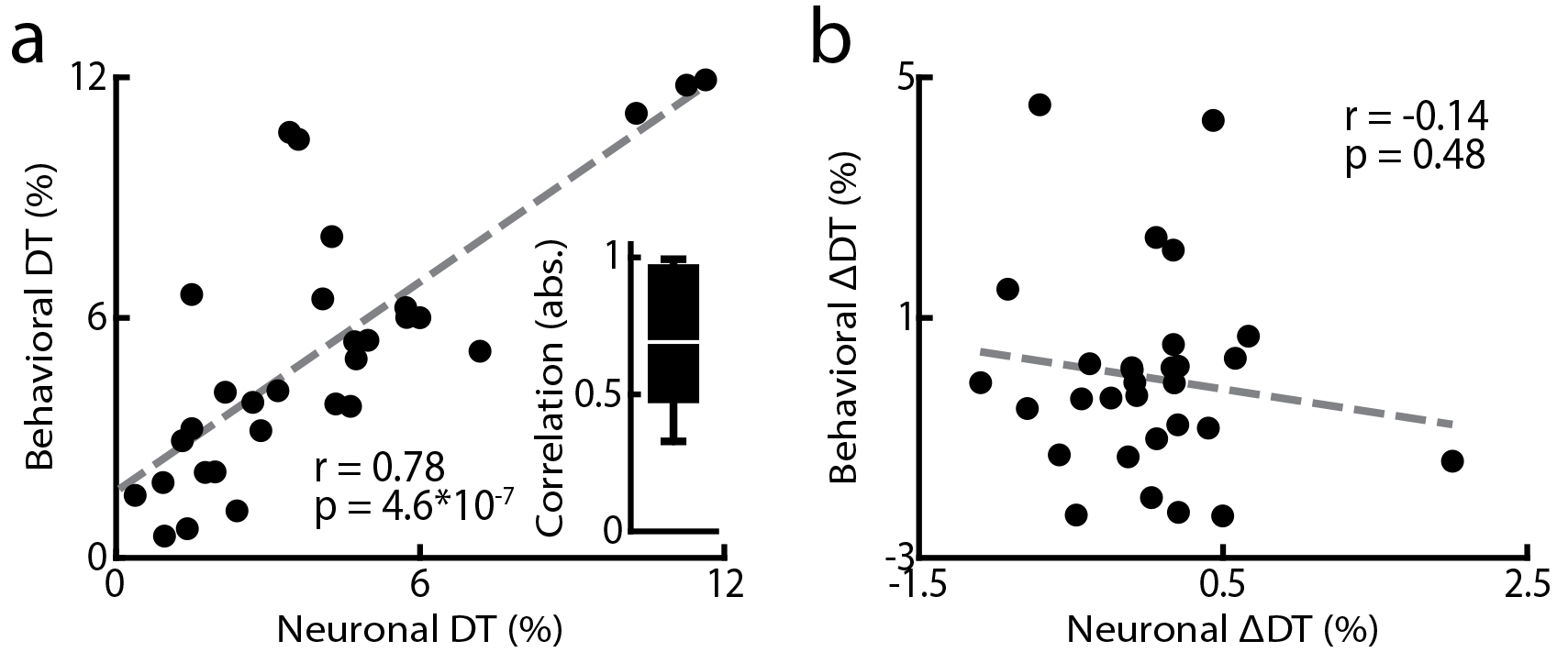

Supplement: S2 Fig — (a) The detection threshold values of PCells and behavior are strongly positively correlated, as indicated by a high r-value (Pearson’s correlation coefficient: r = 0.93; p = 4.6 × 10^−7^) The inset shows a whisker box of the correlation coefficient obtained for each pair. (b) The residuals of neuronal and behavioral detection threshold values obtained for repetitive stimulation (3 repetitions) were not significantly correlated (Pearson’s correlation coefficient: r = −0.14, p = 0.48). The data can be downloaded at https://figshare.com/s/93707200732db87bb80f. PCell, pyramidal cell. (TIF) [file pbio.2005239.s002.tif]

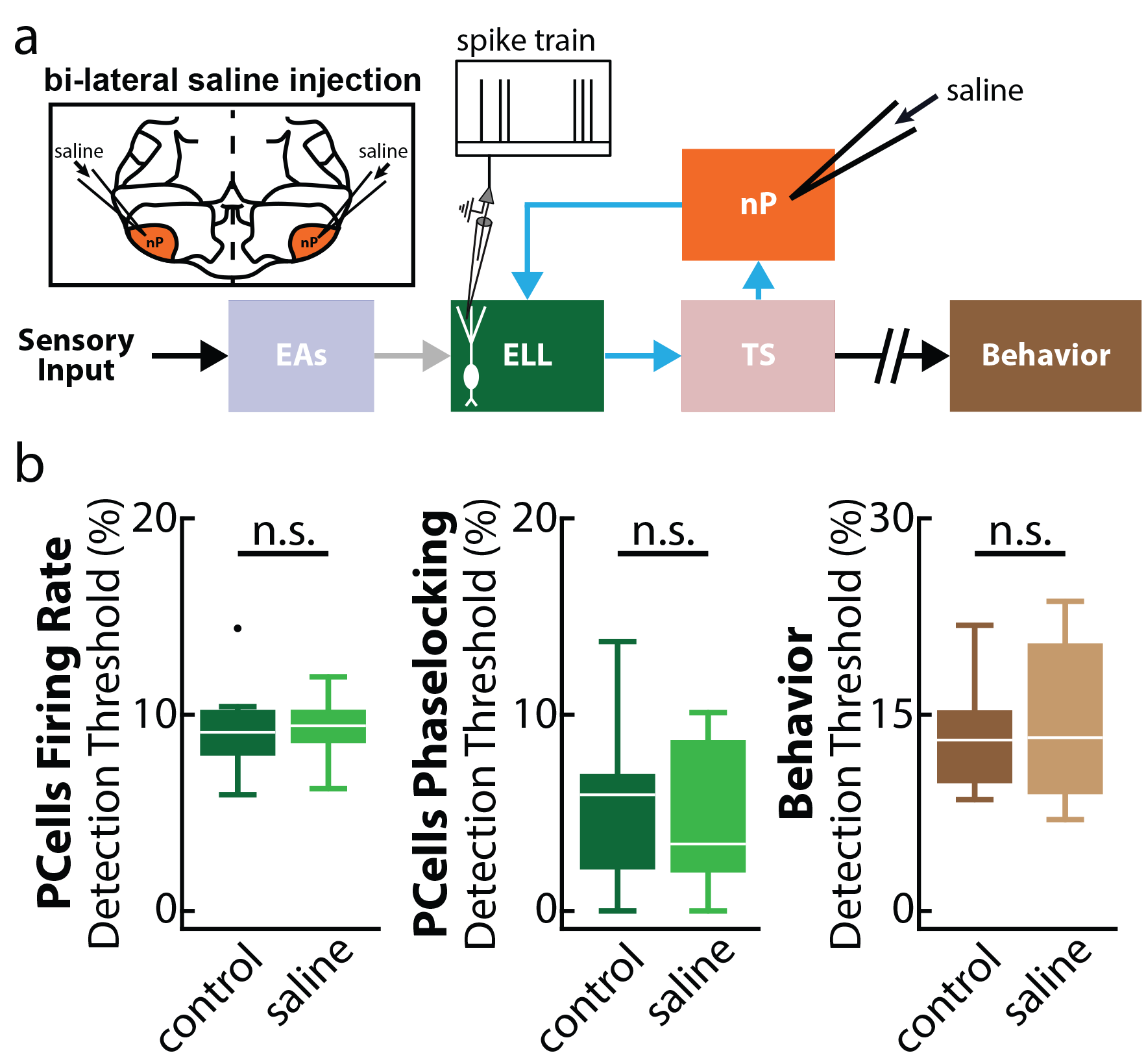

Supplement: S3 Fig — (a) Relevant anatomy diagram showing the main brain areas considered. Recordings were made from individual PCells. (b) Left: PCell firing rate detection threshold values did not change after saline injection (control: 9.4% ± 1.0%, saline: 9.3% ± 0.7%; Wilcoxon sign rank test, n = 7; p = 0.94). Middle: PCell VS detection threshold values did not change after saline injection (control: 5.4% ± 1.7%, saline: 4.8% ± 1.5%; Wilcoxon sign rank test, n = 7; p = 0.59). Right: Behavioral detection threshold values did not change after saline injection (control: 13.9% ± 1.5%, saline: 14.1% ± 1.9%; Wilcoxon sign rank test, n = 10; p = 0.99). “ns” indicates no significant difference. The data can be downloaded at https://figshare.com/s/93707200732db87bb80f. nP, nucleus praeeminentialis; PCell, pyramidal cell; VS, vector strength. (TIF) [file pbio.2005239.s003.tif]

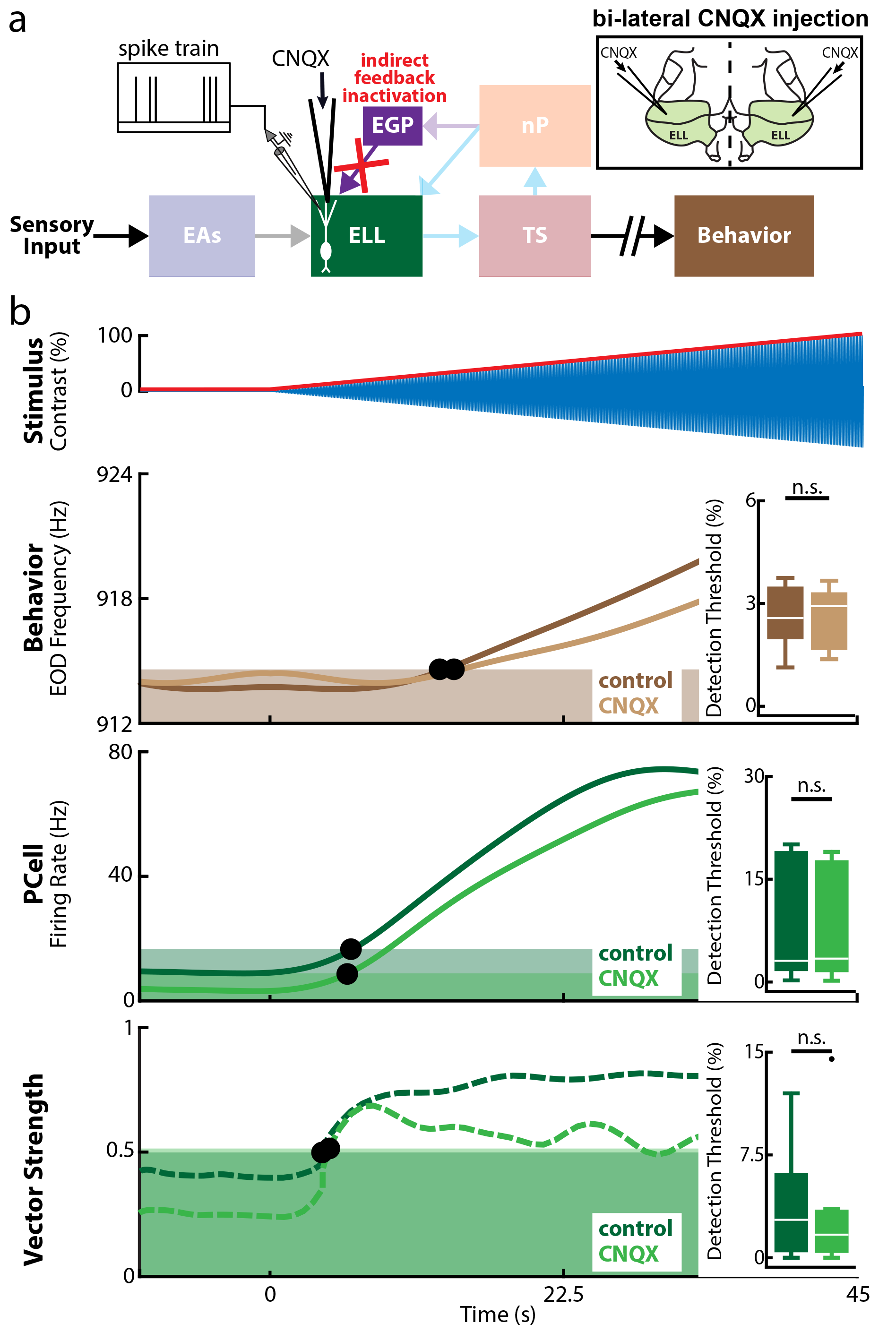

Supplement: S4 Fig — (a) Relevant anatomy diagram showing the main brain areas considered. Recordings were made from individual PCells. Right inset: bilateral injection of the non-NMDA glutamate receptor antagonist CNQX near the apical dendrites of ELL PCells in the molecular layer near the cell being recorded from, as well as to test the effects on behavior. (b) Top: Example behavioral responses to increasing contrast (top) before (dark brown) and after (light brown) bilateral CNQX injection. Middle: Example firing rate responses to increasing contrast from an example ELL pyramidal neuron (dark green) and after (light green) bilateral CNQX injection. Bottom: Example time-varying VS responses to increasing contrast from the same ELL pyramidal neuron (dark green) and after (light green) bilateral CNQX injection. We found that both behavioral (top, inset, control: 2.6% ± 0.4%; CNQX: 2.5% ± 0.4%, Wilcoxon sign rank test, n = 7, p = 0.81) and neural (firing rate: middle inset, control: 8.5% ± 3.2%; CNQX: 8.1% ± 3.0%, Wilcoxon sign rank test, n = 8, p = 0.31; VS: bottom inset, control: 3.8% ± 1.5%; CNQX: 3.2% ± 1.7%, Wilcoxon sign rank test, n = 8, p = 0.55) detection thresholds were not affected by CNQX injections. Note that previous studies have shown that saline injection within the molecular layer does not affect behavioral responses [23,76,113]. As a positive control, we note that injection of CNQX significantly decreased the baseline (i.e., in the absence of stimulation) firing rates of ELL PCells (control: 12.75 ± 1.98 spk s−1; CNQX: 6.79 ± 1.18 spk s−1, Wilcoxon sign rank test, n = 8, N = 3 fish, p = 0.0078), which is consistent with previous results[55,114]. “ns” indicates no significant difference. The data can be downloaded at https://figshare.com/s/93707200732db87bb80f. CNQX, 6-cyano-7-nitroquinoxaline-2,3-dione; ELL, electrosensory lateral line lobe; NMDA, N-methyl-D-aspartate; PCell, pyramidal cell; VS, vector strength. (TIF) [file pbio.2005239.s004.tif]

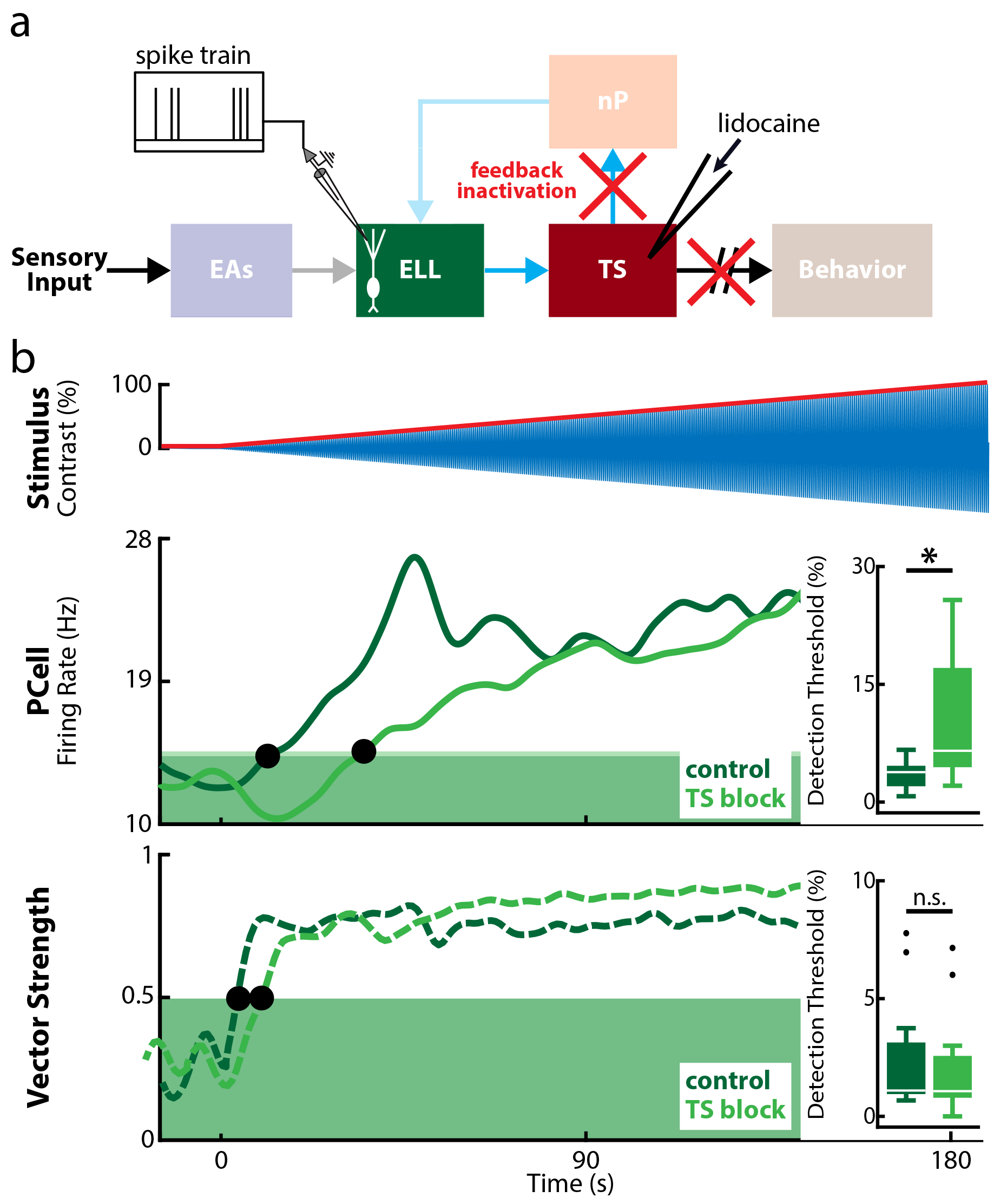

Supplement: S5 Fig — (a) Relevant anatomy diagram showing the main brain areas considered. Lidocaine was injected in TS while recordings were made from individual PCells within the contralateral ELL. (b) Top: Example firing rate responses to increasing contrast (top) from an example ELL pyramidal neuron (dark green) and after (light green) lidocaine injection. Bottom: Example time-varying VS responses to increasing contrast (top) from the same ELL pyramidal neuron (dark green) and after (light green) unilateral lidocaine injection into the contralateral TS. We found that firing rate detection thresholds significantly increased after lidocaine application (middle inset, control: 3.6% ± 0.5%; lidocaine: 13.6% ± 4.9%, Wilcoxon sign rank test, n = 12, N = 5 fish, p = 4.88 × 10^−4^). In contrast, VS detection thresholds were not significantly altered by lidocaine injections into the contralateral TS (bottom inset: control: 2.4% ± 0.7%; Lidocaine: 2.1% ± 0.7%, Wilcoxon sign rank test, n = 12, p = 0.42). We note that these results are qualitatively similar to those obtained by injecting lidocaine into nP and thereby blocking STCells (compare with Fig 5). “ns” indicates no significant difference. The data can be downloaded at https://figshare.com/s/93707200732db87bb80f. ELL, electrosensory lateral line lobe; nP, nuclus praeeminentialis; PCell, pyramidal cell; STCell, stellate cell; TS, torus semicircularis; VS, vector strength. (TIF) [file pbio.2005239.s005.tif]

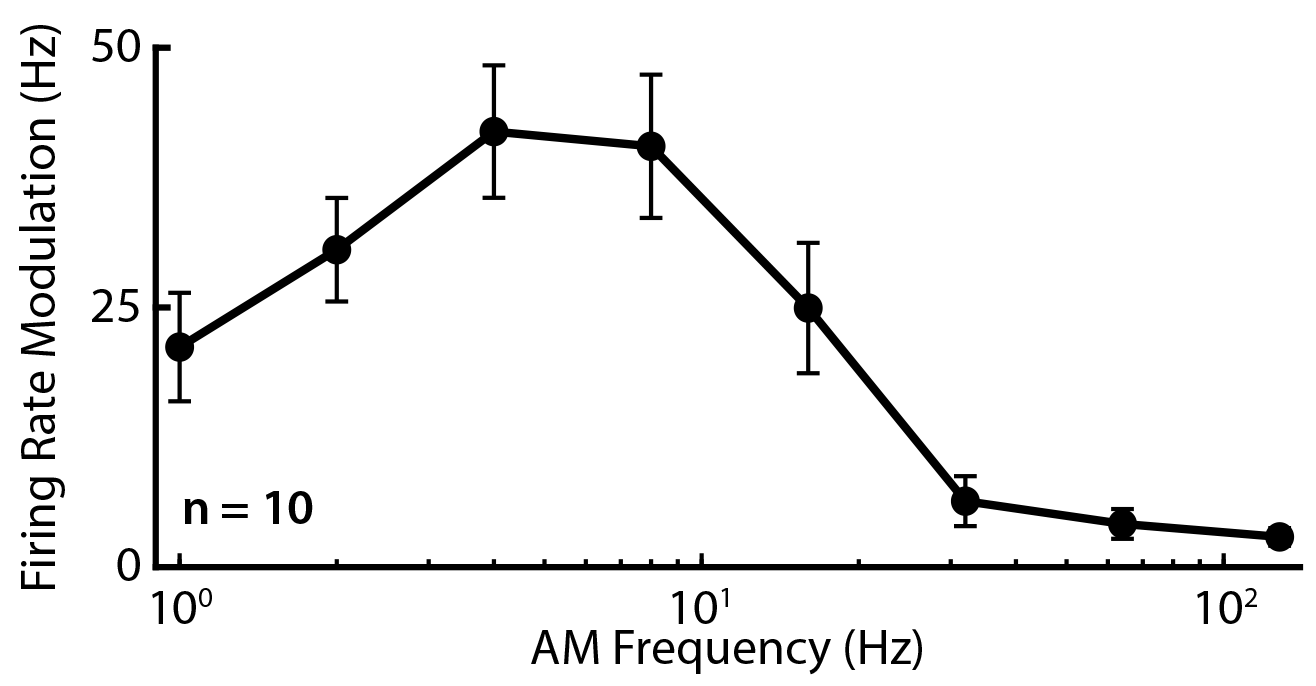

Supplement: S6 Fig — Response profile of our nP STCell population (n = 10) to different sinusoidal AM frequencies. The firing rate modulation peaks around 4–8 Hz and is negligible for AM frequencies >32 Hz. This is similar to that reported previously for STCells [30] and strongly differs from properties of other neuron types within nP [31]. The data can be downloaded at https://figshare.com/s/93707200732db87bb80f. AM, amplitude modulation; nP, nucleus praeeminentialis; STCell, stellate cell (TIF) [file pbio.2005239.s006.tif]

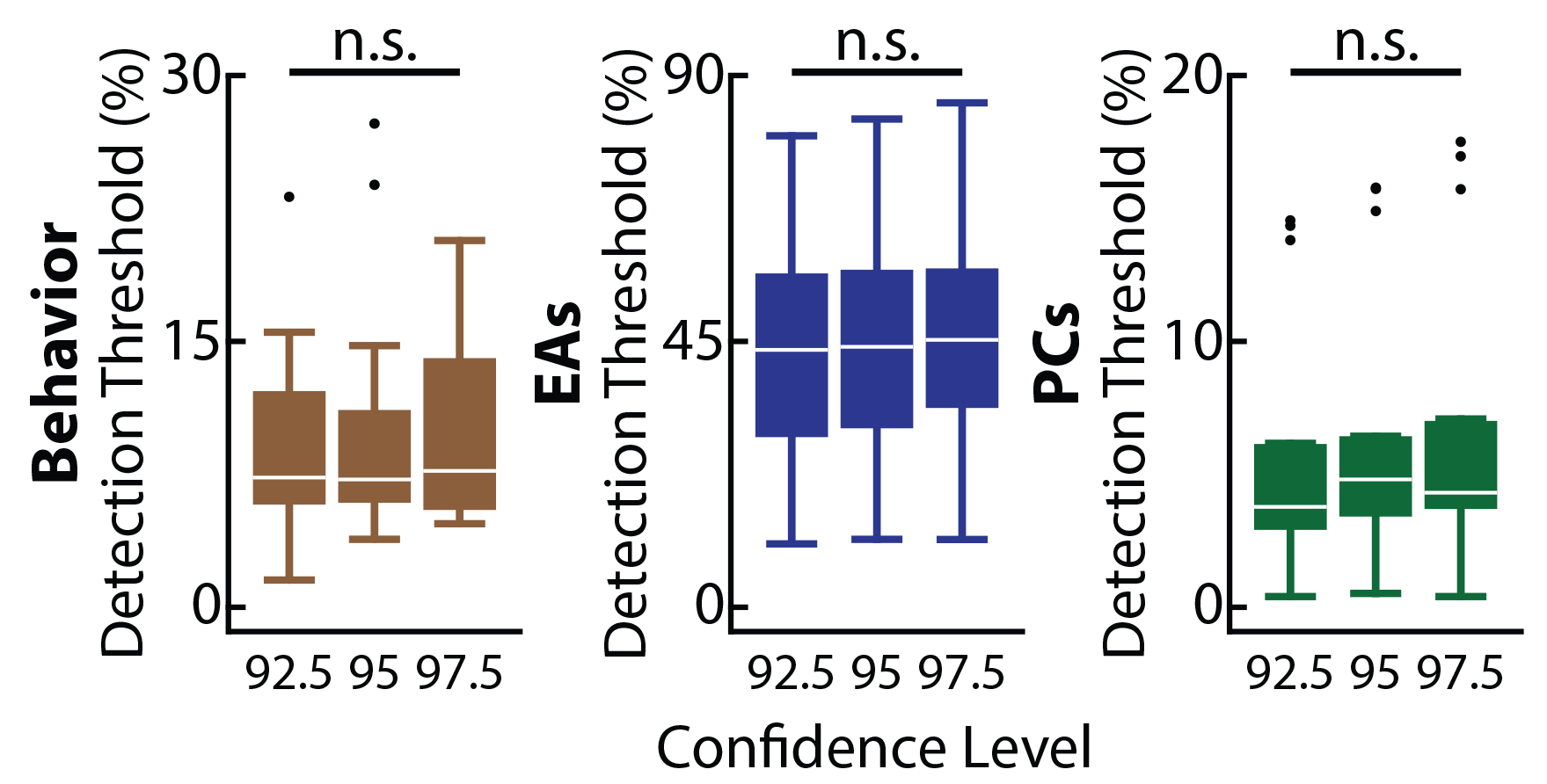

Supplement: S7 Fig — No significant differences were seen for behavior (brown, left), EAs (blue, middle), and PCells (green, right) when altering the significance level (Kruskal-Wallis, df = 2; Behavior: p = 0.99; EAs: p = 0.99; PCells: p > 0.66 with Bonferroni correction). The data can be downloaded at https://figshare.com/s/93707200732db87bb80f. EA, electrosensory afferent; PCell, pyramidal cell. (TIF) [file pbio.2005239.s007.tif]
